# Selection bias on intellectual ability in autism research: a cross-sectional review and meta-analysis

**DOI:** 10.1186/s13229-019-0260-x

**Published:** 2019-03-01

**Authors:** Ginny Russell, William Mandy, Daisy Elliott, Rhianna White, Tom Pittwood, Tamsin Ford

**Affiliations:** 1 College House, University of Exeter Medical School, University of Exeter, Exeter, EX1 2LU UK; 2 UCL Research Department of Clinical, Educational and Health Psychology, Gower Street, London, WC1E 6BT UK; 3 College of Social Science and International Studies, Byrne House, University of Exeter, Exeter, EX4 4PJ UK; 4 Brain in Hand, Innovations Centre, University of Exeter, Exeter, EX4 4QJ UK

**Keywords:** Selection bias, Autism, Intellectual disability, Autism spectrum disorder, Nosology

## Abstract

**Background:**

Current global estimates suggest the proportion of the population with autism spectrum disorder (ASD) who have intellectual disability (ID) is approximately 50%. Our objective was to ascertain the existence of selection bias due to under-inclusion of populations with ID across all fields of autism research. A sub-goal was to evaluate inconsistencies in reporting of findings.

**Methods:**

This review covers all original research published in 2016 in autism-specific journals with an impact factor greater than 3. Across 301 included studies, 100,245 participants had ASD. A random effects meta-analysis was used to estimate the proportion of participants without ID. Selection bias was defined as where more than 75% of participants did not have ID.

**Results:**

Meta-analysis estimated 94% of all participants identified as being on the autism spectrum in the studies reviewed did not have ID (95% CI 0.91–0.97). Eight out of ten studies demonstrated selection bias against participants with ID. The reporting of participant characteristics was generally poor: information about participants’ intellectual ability was absent in 38% of studies (*n* = 114). Where there was selection bias on ID, only 31% of studies mentioned lack of generalisability as a limitation.

**Conclusions:**

We found selection bias against ID throughout all fields of autism research. We recommend transparent reporting about ID and strategies for inclusion for this much marginalised group.

**Electronic supplementary material:**

The online version of this article (10.1186/s13229-019-0260-x) contains supplementary material, which is available to authorized users.

## Introduction

Selection bias occurs when representation of the population of interest, in this case people with autism spectrum disorder (ASD), is not achieved on key characteristics. Studies are vulnerable to selection and recruitment bias when those with particular characteristics are excluded or under-recruited [1]. Selection bias occurs when samples are drawn from non-random sub-populations to estimate what is happening in the whole population, causing errors in the effect size estimation. We set out to review whether participants with intellectual disability (ID) are excluded or routinely under-recruited in studies that feature people on the autism spectrum as the population of interest. The only other study we know of to address this topic is an annual research review that reported bias on ID to be common in psychiatric neuroscience, but the focus was limited to studies of neuroimaging [2].

The inclusion of participants with ID in studies of autism is important because our knowledge base about autism is predicated on the assumption that what we know about autism can be applied to all people with autism. So for example, in the case of research about autism interventions, if selection bias against participants with ID occurs, it will mean that interventions that have been shown to improve outcomes may not be effective in the population with ID.

To give a more concrete example, consider an autism intervention trial that tests a parent-delivered behavioural intervention on children, with severity of autism symptoms as a primary outcome. If data on the proportion of autistic children with ID are not reported in either the initial trial or the long-term follow-up, then it would not be clear if there was selection bias on ID. If there is selection bias on ID, however, the evidence for the effectiveness of this intervention in the ID group would be lacking. In this scenario, interventions, which are often somewhat intensive and parent-mediated, may be erroneously recommended to parents already stretched by having a child with challenging intellectual difficulties.

Mounting evidence suggests sub-groups on the autism spectrum may have distinct aetiology, as well as varied responses to interventions, treatment and diverse care needs [3–5]. The argument above can be applied to any autism research that focusses on the aetiology and developmental pathways that lead to autistic symptoms and traits. If our knowledge about autism genetics, for example, is predicated on a sample primarily drawn from children without ID, this may mean that genetic predispositions to autism with ID are not correctly understood. If such biases are not transparently reported and acknowledged in citing literature, the implications may be that the autism literature erroneously understands the ‘genetics of autism’ which in fact best explains cases without intellectual disability. Thus, our entire knowledge base about autism rests on an assumption that selection bias is not endemic in the research literature. Lack of evidence for autistic children with ID is particularly concerning in the case of drug development because parents of children with autism who have more profound impairment are likely to be the most enthusiastic users of such treatments [6].

Of course, there are some instances of autism research where exclusion of people with ID is scientifically necessary. These include studies of the specific needs and capacities of autistic people without ID, for example, investigating their experiences in a university or employment within the technology sector. In most cases, however, strategies should be taken to ensure meaningful inclusion of participants with ID is achieved [7].

Historically, 70–75% of autistic children were estimated to have ID [8]. This figure has fallen, and recent estimates suggest globally 50–55% have an IQ below 70 [9, 10]. This percentage is currently considered to be the most reliable for the current prevalence. The historical shift in the USA from a predominantly ‘lower functioning’ sample to a ‘higher functioning’ one has been documented in a sequential cohort study of over six million children from California published in 2012 [11]. This demonstrated the chances of autism diagnosis were 15 times greater for ‘high-functioning’ children in 2002 compared to 1992, whereas odds of diagnosis increased only fourfold for the ‘lower functioning’ group. Clearly, the prevalence of the proportion of autistic individuals with ID is a moving target as more individuals without ID are being identified today than previously [11]. Nevertheless, according to a worldwide systematic review, the autism population is still ‘associated with intellectual impairment among a large and significant portion of those affected’ [12].

We hypothesised that many studies that are presented as including people from the entire autism spectrum are actually based on the findings drawn from predominantly non-ID samples. Thus, our primary objective was to assess if there was a selection bias by ID through all sub-fields of autism research. A secondary objective was to record the quality of reporting in studies concerning potential selection bias.

## Methods

We aimed to establish if all fields of autism research exhibit selection bias. We utilised an established review methodology [13] which provided a cross-sectional sample across 1 years’ autism research publications.

### Sampling strategy

**Fig. 1:**
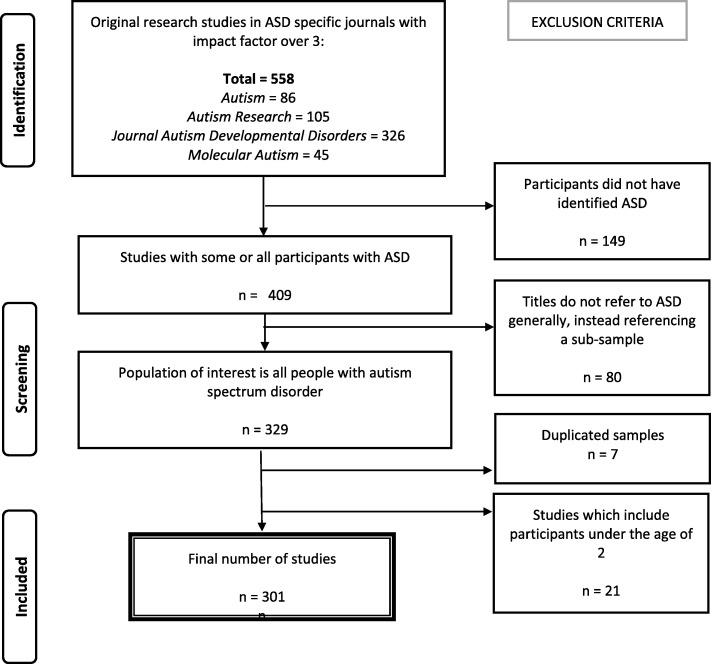
Search and sampling strategy

Figure 1 illustrates the sampling strategy for studies. To sample a representative cross-section of strong and influential autism research studies, we reviewed all studies published in autism-specific journals with an impact factor higher than 3 between 1 January 2016 and 31 December 2016 (dates for printed, as opposed to online publication). The journals that met these criteria were *Molecular Autism*, *Autism Research*, *Journal of Autism and Developmental Disorders*, and *Autism: International Journal of Research and Practice*. All studies published in these journals were eligible for our sample. The methodology was adapted from Lee and colleagues [13]: like them, we developed a sampling protocol that is easily replicable. We wanted to sample the literature at one time point as estimates of the proportion of autistic individuals with ID have shifted over time. A cross-sectional method of review (over 1 year) was therefore best placed to test our hypothesis.

It is important to emphasise that we did not set out to undertake a full systematic review, although we utilised systematic review methodology in terms of screening titles, data extraction techniques and meta-analysis to investigate the presence and extent of selection bias among research studies involving participants with autism.

### Inclusion and exclusion criteria

To be eligible, papers needed to report empirical research, rather than commentaries, letters, editorials, reviews and errata. We included data-based short reports and original research articles that covered children or adults identified as having ASD. We aimed to provide a cross-section of research studies across the field where titles of published studies stated the population of interest was people on the autism spectrum. In order to test for selection bias, we defined *population of interest* according to that stated in article titles. Articles were therefore included where the titles referred to ‘autism’, ‘autism spectrum disorders’ or ‘autism spectrum conditions’, i.e. stated population of interest was the entire autism spectrum, and findings were applicable to this. We therefore excluded research studies whose titles stated the population of interest were sub-groups of the autism spectrum in terms of lower/higher cognitive functioning. Examples that were excluded had titles referring to ‘high-functioning’ or ‘minimally verbal’ groups as their findings only applied to these groups. Included articles were those where at least a proportion of the study sample was identified as having ASD, regardless of the study design. We excluded studies where part of the sample or the entire sample was in infancy (age below 2) because this is before autism can be reliably identified [14]. Also, IQ measurement is not possible in this group.

The process of screening for inclusion was carried out by two researchers (DE, RW), who independently screened titles and abstracts. Disagreements and uncertainties were resolved by a discussion at weekly meetings (with GR and TP) to resolve any uncertainties. For those records deemed relevant after the title and abstract screening, full-text articles were retrieved. Articles were stored as full-text references using Zotero software.

### Data extraction

Data were extracted from 20 included studies by 4 researchers to develop a pilot data extraction form. The remaining articles were divided between 3 researchers (RW, DE and TP) who extracted data into the finalised bespoke data extraction form. Over 20% of studies were rated by 2 or more reviewers (*n* = 61). Inter-rater reliability was good to perfect: agreement values for Cohen’s kappa for primary extractor (DE) vs secondary extractor (RW) ranged from 0.72 to 1. The lower range was due to the lack of consensus over sub-field, as some studies belonged to multiple fields, data extractors sometimes coded studies under different sub-fields.

Articles were classified into six sub-fields based on the categories provided by 2008 US funding portfolio analysis (epidemiology; intervention studies; basic biology including genetics, psychology and neuroscience; social and services research; diagnostic scales/identification and screening). After calculating kappa, studies were classified into sub-field by consensus within the weekly team meetings. Data extracted from each published study included the total number of participants and number of participants on the autism spectrum, for the latter their mean age and age range (which we classified as covering children (age 2–10), adolescents, (age 11-18) adults (18+)), gender ratios, whether participants were minimally or non-verbal, exclusion criteria, sub-field of study, recruitment setting and method of case ascertainment.

Data recorded for each included study are presented in Additional file 1: Table S1. Additional file 2 gives a complete citation list.

### Primary objective: assessing selection bias by ID

#### Ascertaining ID

ID is often defined in autism research studies using intelligence quotient (IQ) above and below 70. IQ is used to define both ID [IQ < 70] and high-functioning autism [IQ > 70] and is the most commonly used sub-type in autism research [15, 16]. For each study where data were available, we classified the proportion of the ASD sample that did not have intellectual disability (No ID) using procedures which have been applied by others [9, 17]. The hierarchy below was used so that 1 was used in preference to 2, etc. We recorded the number of studies that did not include any of this information and estimated the proportion of ASD participants in each study that have IQ above 70 (No ID). The proportion of participants without ID was estimated using the first two methods in two thirds of all cases (for included studies).

1. Reported as having IQ above 70, cut-off based on standard accepted ranges of intellectual disability and borderline intellectual disability
2. Estimation of proportion with IQ above 70 taken from the mean and SD of IQ
3. The proportion that was described as ‘high-functioning’
4. The proportion that was identified as not having ID

We did not estimate numbers with ID where data were missing, i.e. when studies did not report on any measures we used to ascertain ID (above). We did not impute data. Where data on ID were missing, we recorded and reported on this.

#### Defining selection bias by ID

We categorised studies where over three quarters of the sample were without an intellectual disability as showing selection bias, as population estimates of levels of ID among autistic people are around 50%. The highest published epidemiological estimate of the prevalence of ASD children without ID is 69%, taken from US parent report [18]. This is likely an overestimate due to a self-selecting sample. Nevertheless, to err on the side of caution, we defined selection bias with a conservative approach (a large bias where 75% or more of participants in each study had no ID).

#### Meta-analysis

We used random effects meta-analysis to estimate the total proportion of all ASD participants who did not have ID across all the included studies (where data were provided). This was achieved by weighting the estimate by the number of ASD participants in each study using the *metaprop* command on Stata 15 software. We then estimated the mean IQ overall and by sub-field of autism research. We also estimated the overall proportion of non- and minimally verbal autistic participants, where these data were provided. As a sensitivity analysis, we meta-analysed studies that had not actively excluded on ID.

### Secondary objective: assessing quality of reporting within and between studies

We wanted to find out about the quality of reporting as well as selection bias. We recorded the number of included studies that did not report any data on participants’ intellectual dis/ability according to the definition above. We also recorded where studies that showed selection bias on ID did not report a lack of generalisability as a limitation or mention sub-populations in their abstracts.

For included studies that did not contain any participants with intellectual disability (100% No ID, *N* = 78), forward citation chasing was carried out to establish whether later publications cited the studies as a source of knowledge about all people with autism or took the lack of generalisability into account. ‘Forward citation chasing’ refers to the method of finding articles that have cited a previously published work. Cited reference searching was carried out in Google Scholar, which indexes citing studies for each paper. The first three published citing articles were selected where more than three were available for one study. This method of tracking citations is simply another way of searching databases to find relevant sources and articles and has been used in multiple reviews (e.g. [19, 20]).

## Results

There were 563 eligible articles, in total, of which 301 studies were included (Fig. 1).

There were 7,215,166 participants across the 301 included studies, of which 100,245 participants were identified as having ASD. The bulk of the non-autistic participants came from a few very large population-based studies, with one cohort accounting for over six million participants [21]. Additional file 1: Table S1 shows attributes of individual studies including the method of case ascertainment. Table 1 shows the median number of participants for each type of study. Overall, the median number of participants identified as having ASD across the 301 studies was 32. On average, four fifths of the participants in each study were male.

**Table 1:** Descriptive statistics for studies and participant groups by field

Field	Studies, *N* (%)	Total number of participants		Number with ASD		Characteristics of ASD population	
		Median	IQR	Median	IQR	Mean % male	Mean age (years)
Interventions	35 (11)	33	70	30	48	82	11
Biology—neuroimaging, neuropathology, genetics and omics	38 (13)	51	69	26	41	83	17
Epidemiology	22 (7)	10,197	54,362	457	1623	72	8
Psychology and cognitive neuroscience	126 (42)	54	49	24	25	82	17
Social, school, education and family circumstances	45 (15)	97	198	74	123	78	14
Diagnostics, diagnostic scales, identification, screening and scale development	35 (12)	162	298	103	197	79	15
All	301 (100)	62	116	32	67	80	15

Between disciplines, unsurprisingly, epidemiological studies had the highest number of participants, and basic biological studies had the lowest number. Seventy-nine percent of studies covered children, adolescents or both. Only two studies recruited adults where the youngest participant was over 25. Thus, the majority of ASD research was conducted with children and adolescents, with few studies focussing on older adults.

### Selection bias on ID

**Fig. 2:**
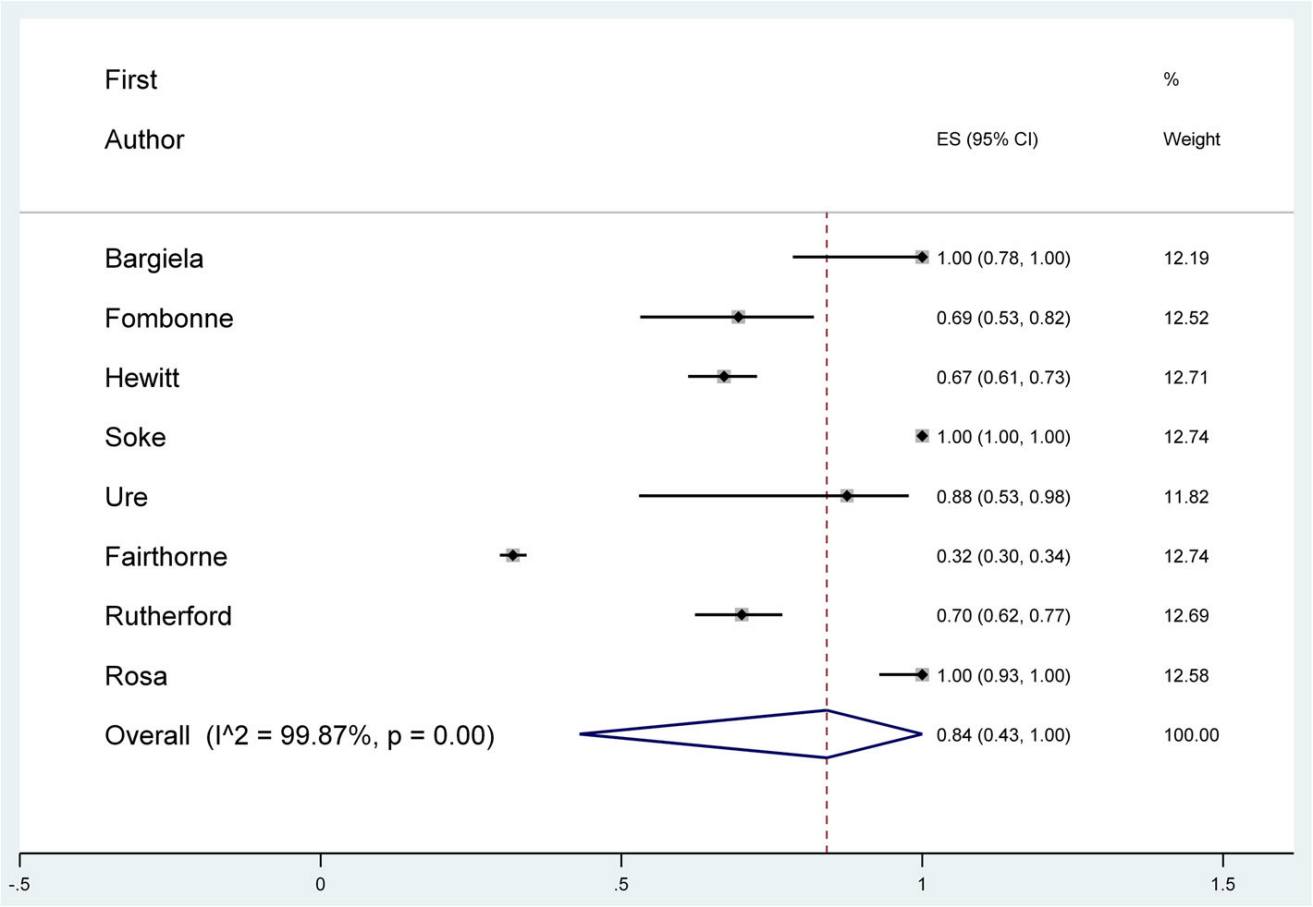
Forest plot for meta-analysis of studies in epidemiology

The majority (82%) of the 165 studies that provided IQ/ID data showed selection bias against individuals with ID (Table 2) despite declaring the population of interest to be, and applying their findings to, the entire autism spectrum. Column 5 of Table 2 shows that an estimated 94% of all ASD participants from 165 studies that provided data did not have ID: the random pooled effect was 0.94 (95% CI 0.94–0.97). Pooling data from all studies where IQ was provided, the mean IQ was 93.8 (*n* = 136). Figure 2 shows the forest plot for studies in epidemiology, which was the field with the lowest bias. The random pooled effect (given as the effect size in Stata output) is illustrated by the dotted line in Fig. 2.

**Table 2:** Selection bias in ASD research on intellectual dis/ability for studies that reported data (*n* = 165)

Field	Total *N*	Studies where over 75% of the sample had No ID		Studies where the entire sample had No ID		Estimated % of ASD participants without ID^a^ (95% CI)
		*N*	%	*N*	%	
Intervention	12	8	67	5	42	87 (68, 99)
Biology	24	22	92	14	58	97 (92, 1.00)
Epidemiology	8	4	50	2	25	84 (43, 100)
Psychology	78	68	87	45	57	97 (94, 99)
Social	23	8	80	9	39	87 (80, 93)
Diagnosis	20	19	83	4	20	84 (82, 94)
Overall	165	136	82	79	48	94 (91, 97)

^a^Calculated using meta-analysis (i.e. weighted by *N* per study)

We also estimated the percentage of participants who were non- or minimally verbal using meta-analysis of the 50 studies that reported data: the random pooled effect was 0.98 (95% CI 0.93–1.0). Thus, we estimated that 94% of autistic participants in the included studies did not have ID (an estimated 6% had ID) and only 2% of participants were non- or minimally verbal. The random pooled effect size in sensitivity analysis removing studies that had deliberately excluded participants with ID was 0.89 (95% CI 0.84–0.94).

### Reporting quality within and between studies

Out of 301 included studies, most (55%, *n* = 165) did provide ID data. A quarter, 25% (*n* = 77), of studies specifically excluded autistic participants with intellectual disability as defined by our hierarchy. Over a third, 38% (*n* = 114), did not report any data on ID status according to our measures or exclude participants with ID, although some of these did report on rarer measures of developmental delay, especially studies of younger children. Only a small proportion of included studies reported data on the proportion of ASD participants who were verbal or non-verbal/minimally verbal: just 50 (17%) of the 301 studies.

One hundred and thirty-six of the 165 included studies that did provide ID data showed selection bias against participants with ID (Table 2). The majority of these studies (52%) did not report sub-populations in their abstracts or lack of generalisability (*n* = 71). Only 31% mentioned a lack of generalisability or selection bias as a limitation to the study. There was a wide variation in quality in the way the lack of generalisability was presented for those that did refer to it, ranging from simply stating the participant characteristics, e.g. ‘we only looked at higher functioning children’ [22], to meticulous consideration of the potential for biases, ‘we acknowledge several limitations to this study. Data were collected through a specialist clinical diagnostic service for ASD, where adults are expected to have IQ in the normal range. Thus the sample does not reflect the ASD community as a whole’ [23].

#### Forward citation chasing

For studies that did not include any participants with ID, we checked 187 later publications that cited the 2016 findings. Ninety-one percent of the citations cited the 2016 results as being about autism generally or applicable to all people with autism, despite being drawn from studies where people with ID did not appear in the sample. Therefore, as we suspected, studies’ findings are typically generalised to the entire spectrum despite being exclusively drawn from the intellectually able group.

## Discussion

### Substantive findings

In order to learn about selection bias on ID in autism research, we sampled all papers published in the most cited specialist autism journals in 2016. First, the review found considerable evidence of selection bias on ID: participants with ID were under-recruited to all sub-fields of autism research. Second, the reporting of participant characteristics was generally poor, with information about intellectual ability and/or ID often absent. There was a limited discussion of this, and forward citation chasing showed that citing authors did not typically acknowledge bias.

Our findings are consistent with the review that focused on brain imaging studies of autism, which found people with ID were under-represented in neuroimaging studies of autism [2]. It also tallies with individual reports: for example, the US National Database of Autism Research has 47,400 participants, but only 11% have borderline ID or ID (IQ < 85) [24]. Our finding is important because the validity of findings in individual studies is grounded on the assumption that the recruitment of study participants is not overly influenced by selection bias.

For studies using samples without any ID participants, forward citation chasing showed later papers typically cited them to describe knowledge about the whole autism spectrum, without taking sample profiles into account. Selection bias in participant characteristics might mean much of the information we have about autism may not be generalisable to all those with autism. Forward citation chasing illustrated how ‘facts’ travel based on titles and abstracts alone. Only a minority of studies displaying bias mentioned lack of generalisability as a limitation, none referred to sub-samples in their titles. Lack of generalisability was dealt with by some studies in their limitations sections (e.g. [25–28]) but sometimes in a cursory way, although others were examples of best practice. We recommend that if a No ID sample is used, this should be clearly stated in the title and abstract.

### Barriers to participation, and solutions

Selection bias on intellectual disability may occur in autism research because children and adults with more severe difficulties are harder to recruit and retain in research studies and, similarly, that families of children with ID are harder to recruit as they may have less time and resources [29]. There is also an issue around mental capacity to consent to take part in research for some severely affected individuals with ID, so for ethical reasons, severely impaired individuals are excluded. ID is also a spectrum, however, and the lack of capacity to consent to research does not apply to everyone with ID. Another reason for bias is that the field is lacking good instruments to study people with intellectual disabilities [30].

Many research studies in the field of ID have examined strategies to make research more inclusive [7, 31–35], and there are reports and guides full of practical strategies and references to other work [36–38]. Two useful systematic reviews [39, 40] describe the barriers to cognitively disabled individual participation in trials and public health, and another review summarises the last 20 years of research in this area [7]. Such work concludes that strategies to counter selection bias on ID require more time, effort and funding to be put into recruitment. Strategies include one-to-one meetings with participants to explain study aims, enrolment and protocols [31]; piloting and adaptation of measures and working closely with gatekeepers (like service providers) to recruit potential participants. Recruitment phases for populations with ID should be longer than other populations, with researchers prepared to make home visits and visit after hours to allow for time constraints of participants and carers [32]. Being kept up-to-date with the progress of the research projects was found to be an important part of engendering a sense of personal benefit for participants [38].

Many carers experience overwhelming workloads and exhaustion [41]. Carers (most often women [42]) often have little time available for research or resources to support participation in research activities. Respite care for participants to give carers time off could be a strong motivation to enable participation. Participants and intermediaries need motivators to participate in research. A small payment to cover time and travel can help, although can be viewed as coercive [33]. People with ID living independently may also be recruited through peer networks or advocacy movements (e.g. the ‘Academic Autism Spectrum Partnership in Research and Education’ involves self-advocates). A strong self-advocacy movement has been identified as one of the conditions necessary for inclusive research to flourish [43].

Obtaining consent for participants with ID can be challenging and may require another person to give consent on their behalf. This means going through gatekeepers at services organisations, which should start at the highest level [32]. Identification of key workers within the organisations who may become allies to assist researchers in recruitment is a helpful strategy [32].

There is a sub-group of autistic individuals who may not be fluent verbally but are very able with technology to express their needs and wants. Measures to allow them access to research are developing rapidly as technology improves, for example measuring diet through participants’ mobile photos of their food [44]. There are many more people on the spectrum with ID who do not speak but can use such devices and who can, therefore, make their consent and needs for participating known. Although many measures are not currently designed for the population with very severe ID (such as standard IQ tests), there are creative ways to adapt measures and replicate similar constructs but using non-verbal tasks, such as tests of spatial intelligence or Adaptive Behaviour Scales [45].

### Impact of bias by ID

One consequence of the bias encountered is the lack of an evidence base for effectiveness or otherwise of interventions, which have not specifically been trialled in autistic individuals with intellectual disability. This has implications for the introduction of a medication or a specific behavioural therapy or new service. Forward citation chasing indicated that cognitive, psychological and neuroscientific models of autism may be misapplied to the ID group in ongoing research, when in reality, there is no strong evidence base to support their application. Similarly, a diagnostic test that has not been adequately tested on the full population may under-identify the autistic population with ID.

Not only does selection bias lead to inappropriate generalisation, but it may shift the boundaries around who is included for an autism diagnosis. The evidence base about a particular diagnostic category feeds back into what we understand about it via revisions to diagnostic criteria. Thus, the parameters of the category itself are shifted if the research base predominantly contains persons of one type. Autism is a case in point of such ‘looping’, as it was once understood to be a condition affecting very severely impaired children, but is now increasingly associated with non-ID individuals, including adults. Conceptually, autism can be thought of as a collection of multi-dimensional traits that interact with each other and the environment, and these traits may alter with development. They are bounded together as a diagnostic category based on the best current evidence but also bounded for historical, pragmatic and political reasons [46, 47]. If autism research is predominantly drawn from studies of individuals without ID, this will feedback into the evidence base and remodel how we understand ‘autism’. This argument is applicable to all psychiatric diagnoses, with our findings specifically drawing attention where intellectual disability is comorbid [48]. ADHD, psychotic disorder, anxiety disorder and depression all co-occur with ID. Future research could examine if such a bias exists more widely across other psychiatric classes.

### Strengths and limitations

Our review has a number of limitations. First, not all autism research was sampled; we are aware that the studies that include individuals with autism might also appear in a variety of other types of journals that are not specific to autism. These include the highest impact journals (e.g. *Nature*). A systematic review was impractical as an initial scoping review threw up 30,000 hits. Therefore, we simply did not have the capacity to mount a systematic review that was an exhaustive study of all papers relating to autism. This would have included the journals that are specifically about ID (which we would expect to be more balanced for ID and perhaps more transparent in reporting).

A number of studies in our review excluded participants with ID, even though this was not apparent from title screening. Morett et al. [49], for example, operated an exclusion cut-off at 1 SD below mean IQ (IQ < 85). However, even when we removed all studies that deliberately excluded participants with ID, our sensitivity analysis still revealed considerable bias, estimating a large preponderance of autistic participants without ID.

In 2013, the Diagnostic and Statistical Manual Volume 5 (DSM-5) unified the diagnoses of autistic disorder, Asperger’s disorder and Pervasive Developmental Disorder Not Otherwise Specified (PDD-NOS) into ‘autism spectrum disorder’. Our findings may partly reflect the response of the research community to this change. It could be that selection bias towards autistic individuals with an IQ in the normal range is a relatively recent phenomenon, as historically, estimates of population of autistic people with ID were higher. Here, we were interested in sampling the up-to-date (post-DSM-5) picture. Further, it was not possible to report more detail on bias by IQ or by severity of ID as a continuous measure as not enough studies provided data. Nevertheless, this review is the first (we know of) to comprehensively examine a cross-section of autism research for bias by ID in a large sample.

### Recommendations

Our recommendation is to address barriers to research participation for the ID group. We agree with Mulhall et al. that studies that have inclusive strategies for recruitment should become a priority for research funders along with that development of inclusive measures [40]. Researchers and academics can help by recommending grants for funding only where broad inclusion strategies to include participants with ID have been considered or are in place. We suggest that exclusionary recruitment strategies are acceptable, and may be necessary for pragmatic reasons, but should be reported in a transparent way in abstracts and titles and justified carefully in funding applications, explaining why the protocol cannot be modified to include those with ID.

### Conclusion

Our study throws attention on a gap in the research literature for autistic people with ID, but the point could be extended to other hard-to-reach populations, such as people with complex comorbid mental health conditions, severe ID or minimally verbal participants. Future research might investigate whether such groups may be largely absent from the evidence base for autism but also neurodevelopmental conditions more widely, suggesting what we know about a condition may largely reflect groups who are easier to access.

The reporting evidenced in our study would be unproblematic if all autistic people shared the same neurological profiles, genetic predisposition, aetiological pathways, cognitive and perceptual differences and responses to intervention, but recent evidence suggests this may not be the case [3, 5, 50]: it is difficult to find a ‘signal’ that is specific to one ‘autism’. There are real-world implications if selection bias on ID occurs when participants’ intellectual ability is associated with the phenomena under study. This may be the case for studies of intervention, aetiology, service use, diagnosis, neuroscience and psychology/cognitive functioning: all the sub-fields examined in this review.

## Additional files

Additional file 1: **Table S1.** Data extracted from each included study is presented in this table. Data extracted included the total number of participants and number of participants with ASD, mean age gender ratios, sub-field of study and method of case ascertainment. (DOCX 86 kb)

Additional file 2: Supplemental citation list of included studies. (DOCX 56 kb)

## Abbreviations

ASD: Autism spectrum disorder
DSM-5: Diagnostic and Statistical Manual Volume 5
HFA: High-functioning autism
ID: Intellectual disability
IQ: Intelligence quotient
No ID: Did not have intellectual disability
PDD-NOS: Pervasive Developmental Disorder Not Otherwise Specified
