# Supplementary material for: Selection bias on intellectual ability in autism research: a cross-sectional review and meta-analysis
Source: Mol Autism. 2019 Mar 1;10:9. doi: 10.1186/s13229-019-0260-x (PMC6397505; doi:10.1186/s13229-019-0260-x)
Supplement: Supplementary file 1 — Table S1. Data extracted from each included study is presented in this table. Data extracted included the total number of participants and number of participants with ASD, mean age gender ratios, sub-field of study and method of case ascertainment. (DOCX 86 kb) [file 13229_2019_260_MOESM1_ESM.pdf]

Additional File 1

Table S1: Data extracted for studies included in review.

1st Author	Journal [1]	Country[2]	Field (discipline)[3]	Number of Participants	Number with an ASD	How identified with ASD[4]	Term used to reference autism in title [5]	Setting [6]
Adams (1)	JADD	USA	Diagnosis	1221	432	1	1	3
Adams (2)	JADD	USA	Diagnosis	54	54	1	1	3
Allen	JADD	UK	Psychology	32	16	2	1	2
Alvares	Autism Research	Australia	Psychology	56	17	.	1	1
Anderson	JADD	UK	Diagnosis	15	15	4	1	5
Anderson	Autism	USA	Social	334	182	.	1	3
Antezana	JADD	USA	Psychology	66	41	4	1	1
Ausderau	Autism Research	USA	Psychology	960	960	.	1	3, 5
Azmitia	JADD	USA	Biology	21	10	3	2	3
Balboni	JADD	Italy	Diagnosis	52	32	4	1	1
Baldwin	Autism	Australia	Social	82	82	1	1	5
Bargiela	JADD	UK	Epidemiology	14	14	1	3	5
Barnard-Brak	Autism Research	USA	Diagnosis	344	253	.	4	3

[1] JADD = Journal of Autism and Developmental Disorders, MA = Molecular Autism

[2] Country of origin of sample or corresponding author if not specified.

[3] Full field names: Diagnosis, diagnostic scales, identification, screening and scale development; Psychology and cognitive neuroscience; Biology – neuroimaging, neuropathology, genetics and omics; Epidemiology; Interventions; Social, school, education and family circumstances

[4] 1 = Clinical diagnosis, 2 = Autism Diagnostic Observation Schedule, 3 = Autism Diagnostic Interview-Revised, 4 = DSM-4, -5, ICD-9 or -10, 5 = AQ cut-off score, 6 = Educational diagnosis or Special Educational Needs (SEN) classification, 7 = Parent Report of Clinical Diagnosis, 8 = Other (including other ASD scales, medical records, various', self-report)

[5] 1 = Autism Spectrum Disorder(s)/ASD **(73%)**, 2 = Autism **(20%)**, 3 = Autism Spectrum Condition(s)/ASC **(3%)**, 4 = Autism Spectrum **(2%)**, 5 = Autistic **(2%)**

[6] Setting where participants recruited from. 1 = hospital/clinic/centre, 2 = school, 3 = database/registry/larger study, 4 = university, 5 = online/families/support groups/community groups/charities

Barnevik Olsson	JADD	Sweden	Interventions	128	128	1	2	1
Bearss	Autism	USA	Interventions	93	45	1	1	.
Bedford	Autism Research	UK	Diagnosis	104	17	4	5	1
Bedford	Autism Research	UK	Psychology	30	16	4	1	3, 2
Begeer	Autism	NL	Social	51	28	.	2	2
Ben-Itzchak	JADD	Israel	Psychology	43	20	1	1	1
Benning	JADD	USA	Psychology	69	35	1	1	.
Benson	Autism Research	UK	Psychology	43	19	1	2	5
Biggs	JADD	USA	Diagnosis	389	232	6	2	5
Bilaver	JADD	USA	Interventions	3104	250	8	1	3
Bishop-Fitzpatrick	JADD	USA	Diagnosis	180	180	7	1	3
Blackmon	Autism Research	USA	Psychology	130	130	4	1	1
Blumberg	Autism	USA	Diagnosis	1607	1420	.	1	3
Bolic Baric	Autism	Sweden	Social	13	10	4	1	3
Bottema-Beutel	JADD	USA	Psychology	28	9	4	3	3
Bottema-Beutel	Autism	USA	Interventions	33	33	.	1	2
Bouvet	JADD	France	Psychology	25	12	8	1	1
Brett	JADD	UK	Diagnosis	2134	2134	7	1	3
Brewer	Autism Research	UK	Psychology	27	14	.	1	.
Brian	Autism	Canada	Diagnosis	67	23	.	1	3
Brooks	JADD	USA	Interventions	41	10	1	1	1
Brosnan	Autism	UK	Psychology	82	26	.	1	2
Brosnan (2)	JADD	UK	Psychology	75	75	4	4	5
Burger-Caplan	Autism	USA	Psychology	80	23	.	1	.
Cage	JADD	UK	Psychology	66	33	2	4	2
Cai	JADD	Australia	Diagnosis	38	23	1	1	4
Campione	JADD	Italy	Biology	20	9	4	1	1

Caplan	JADD	USA	Diagnosis	162	162	1	1	5
Carmo	JADD	Portugal	Psychology	39	19	4	1	3
Cascio	JADD	USA	Psychology	109	33	1	1	3, 5
Chamak	JADD	France	Diagnosis	76	76	7	1	.
Chang	Autism	USA	Psychology	31	31	.	1	2
Chen	JADD	Australia	Social	30	30	4	1	1
Chen	Autism Research	Taiwan	Biology	72	37	.	1	1
Chen	Autism Research	China	Biology	656	312	.	1	3
Chiang	Autism	Taiwan	Interventions	34	34	.	1	1, 5
Chien	Autism Research	Taiwan	Biology	73	37	.	2	4
Chiu	Autism Research	Canada	Biology	28	28	.	1	3
Cholemkery	JADD	Germany	Diagnosis	463	463	4	1	1
Choque Olsson	Autism	Sweden	Social	22	11	4	2	3
Chowdhury	Autism	USA	Diagnosis	242	242	.	1	3
Christiansz	JADD	Australia	Diagnosis	185	126	4	1	1
Connolloy	Autism Research	USA	Epidemiology	40846	503	.	1	1
Conson	JADD	Italy	Psychology	36	18	3	1	.
Cooper	JADD	UK	Psychology	48	24	1	3	3
Corbett	JADD	USA	Interventions	30	30	1	1	.
Coster	Autism	USA	Diagnosis	365	365	.	1	5
Cox	JADD	USA	Psychology	44	17	1	1	3
Crawford	MA	UK	Biology	32	16	1	2	2
Crea	JADD	Australia	Epidemiology	58	16	3	1	3
Cummings	JADD	USA	Epidemiology	91520	8325	1	1	1
Davids	JADD	NL	Psychology	72	36	1	1	1
de Bildt	JADD	NL	Diagnosis	93	38	1	1	.
Dekker	JADD	NL	Interventions	86	86	1	1	1
Demopoulos	Autism Research	USA	Psychology	76	60	.	1	3

Dempsey	JADD	USA	Psychology	2341	2341	1	1	3
DiCriscio	JADD	USA	Psychology	47	19	.	2	3
Dolan	JADD	USA	Interventions	62	58	.	1	5
Doumas	JADD	UK	Psychology	30	15	1	1	5
Durieux	Autism Research	UK	Biology	50	21	.	1	1
Dynia	JADD	USA	Psychology	70	35	6	1	2
Edmiston	JADD	USA	Social	34	21	1	1	1, 2, 4, 5
Ekhlaspour	JADD	USA	Biology	18	9	4	1	.
Ellis Weismer	JADD	USA	Psychology	60	30	2	1	1, 3
Eussen	Autism Research	Netherlands	Psychology	87	63	.	1	1
Eversole	JADD	USA	Social	131	67	7	1	2
Factor	JADD	USA	Psychology	44	44	1	1	5
Fairthorne	Autism	Australia	Epidemiology	272947	1698	.	1	3
Faja	Autism Research	USA	Psychology	66	66	3	1	1, 2, 5
Fernandes (1)	JADD	Sweden	Social	108	108	1	1	1
Fernandes (2)	JADD	Sweden	Social	76	76	1	1	1
Field	JADD	UK	Psychology	124	51	1	1	2, 5
Findon	Autism Research	UK	Diagnosis	224	98	.	1	1, 3, 5
Fiorentino	MA	USA	Biology	54	20	8	1	3
Fisher	Autism	USA	Social	30	30	.	1	3
Fletcher-Watson	Autism	UK	Interventions	54	54	.	2	1
Floris	MA	USA	Biology	118	42	1	2	1, 2
Fombonne	JADD	Mexico	Epidemiology	36	36	1	1	2
Forbes	JADD	UK	Psychology	51	26	1	1	3
Forgeot d'Arc	MA	Canada	Psychology	39	20	1	1	1
Foster	JADD	Canada	Psychology	72	32	4	1	3
Fridenson-Hayo	MA	Israel,	Psychology	113	55	1	2	1, 2, 3, 5

		Britain, Sweden						
Fujioka	MA	Japan	Psychology	56	21	4	1	1
Gevi	MA	Italy	Biology	60	30	4	5	.
Gilson	JADD	USA	Interventions	3	2	6	2	4
Ginevra	Autism Research	Italy	Psychology	269	82	.	2	2
Granich	Autism	Australia	Epidemiology	208	208	.	1	5
Green	JADD	UK	Social	54	16	3	1	5
Griffin	Autism Research	UK	Psychology	56	24	4	1	2, 3, 5
Grove	Autism Research	UK	Diagnosis	267	158	.	2	3, 4
Guy	Autism Research	Canada	Psychology	89	34	.	1	1
Haas	JADD	Australia	Social	167	77	8	1	4, 5
Hagmann	JADD	USA	Psychology	69	16	2	2	2, 4, 5
Haigh	Autism Research	USA	Psychology	34	17	.	2	.
Hanaie	Autism Research	Japan	Biology	39	19	.	1	1
Hannah	JADD	UK	Social	40	20	1	1	.
Happé	JADD	UK	Diagnosis	146	100	6	1	1
Hare	Autism	UK	Interventions	9	9	4	2	5
Harriage	JADD	USA	Interventions	6	3	1	2	1, 5
Harrop	Autism	USA	Psychology	85	85	.	1	.
Havdahl	Autism Research	USA	Diagnosis	389	226	.	1	1
Hayes	JADD	UK	Psychology	30	15	2	1	4
Hecht	Autism Research	USA & Canada	Epidemiology	158	158	.	1	3
Helt	JADD	USA	Psychology	86	43	2	1	2
Hepburn	Autism	USA	Interventions	33	33	1	1	5
Hewitt	JADD	USA	Epidemiology	12329	255	6	1	3
Hiller	Autism	Australia	Diagnosis	152	152	.	1	1, 5

Hoffmann (1)	JADD	Germany	Biology	50	2	4	2	1
Hoffmann (2)	JADD	Germany	Biology	155	78	4	2	3
Holmes	Autism	USA	Social	198	198	.	1	5
Hong	JADD	USA	Social	120	60	.	1	3
Howe	JADD	UK	Social	16	16	1	3	2
Hranilovic	Autism Research	Croatia	Biology	156	90	4	5	1
Hundley	JADD	USA	Psychology	532	532	2	1	3
Hyman	JADD	USA	Interventions	14	14	1	2	1
Ibrahim	JADD	Canada	Biology	40	20	2	1	3
Irvine	JADD	USA	Psychology	46	24	4	2	3
Ishizuka	Autism	Japan	Interventions	6	6	1	1	1
Izuwah	Autism Research	Nigeria	Epidemiology	75	75	.	2	1
Jaime	JADD	USA	Biology	33	16	2	1	3
Jashar	JADD	USA	Diagnosis	281	146	2	2	1
Kaartinen	JADD	Finland	Psychology	36	18	1	1	1
Kalb	JADD	USA	Epidemiology	6416785	15532	4	1	3
Kana	Autism Research	USA	Biology	32	17	3	2	1
Kanduri	Autism Research	Finland	Social	349	80	4	1	5
Karhson	Autism Research	USA	Biology	25	12	2,3	1	5
Kauschke	JADD	Germany	Social	33	22	2	1	1
Keehn	JADD	USA	Psychology	52	22	2	2	.
Keehn	Autism Research	USA	Psychology	64	32	4	2	.
Kern Koegel	JADD	USA	Interventions	3	3	4	1	1
Kinnear	JADD	USA	Social	502	502	4	1	2
Kirby	JADD	USA	Social	1170	1170	6	1	3
Kirkovski	JADD	Australia	Biology	50	27	4	1	.
Kitzerow	Autism Research	Germany	Diagnosis	21	21	2	2	3
Kleinhans	JADD	USA	Biology	52	27	3	2	.

Kleinhans	Autism Research	USA	Biology	53	25	4	1	.
Koolschijn	JADD	NL	Biology	100	51	1	1	3
Kovarski	JADD	France	Psychology	42	20	1	2	1
Kranz	Autism Research	Germany	Social	667	667	4	1	1
Kumazaki	MA	Japan	Psychology	43	20	4	1	1
Lehnhardt	JADD	Germany	Psychology	107	107	1	1	1
Lever	Autism Research	NL	Psychology	236	118	4	1	1
Li	JADD	China	Psychology	60	30	4	1	2
Libero	Autism Research	USA	Social	40	20	3	2	.
Liew	Autism Research	Denmark	Epidemiology	64322	1027	4	1	3
Lim	Autism Research	Oman	Biology	27	15	4	2	1
Liu	Autism Research	Japan & Taiwan	Social	1615	789	4,3	1	5
Locke	Autism	USA	Social	102	51	.	1	3
Luo	Autism Research	USA	Psychology	156	26	4	1	1, 5
Ma	JADD	USA	Psychology	7	4	8	2	3
Macizo	JADD	Spain	Psychology	40	20	4	1	2
Mackie	Autism Research	USA	Psychology	30	15	4	1	1
MacMullin	Autism	Canada & USA	Social	311	139	.	1	5
Mandy	Autism	UK	Interventions	37	37	.	1	2
Mari-Bauset	JADD	Spain	Interventions	105	105	2	1	2
Marini	JADD	Italy	Psychology	154	77	2	1	1
Marler	JADD	USA	Biology	82	82	4	1	3
Mazurek	JADD	USA	Psychology	162	81	2	1	1
McCormick	Autism	USA	Psychology	79	26	.	1	1, 5
McCoy	JADD	USA	Epidemiology	42747	915	7	1	3
McVey	JADD	USA	Interventions	47	47	1	1	1, 5

Mhatre	JADD	India	Social	80	80	1	1	1
Minshawi	MA	USA	Interventions	101	67	4	1	1
Moore	Autism	UK	Psychology	38	19	.	1	2, 5
Morett	JADD	USA	Social	39	18	.	1	.
Mouga	JADD	Portugal	Psychology	445	224	3	4	1
Muller	JADD	Germany	Diagnosis	56	33	2	1	3
Murdaugh	Autism Research	USA	Interventions	45	26	1	2	.
Mutluer	Autism Research	Turkey	Psychology	117	64	1	1	2
Nader	JADD	Canada	Diagnosis	47	25	4	5	3
Neuhaus	Autism Research	USA	Biology	36	18	.	2	3
Newbutt	JADD	USA	Interventions	29	29	.	2	5
Nguyen	MA	France	Biology	14	8	1	2	.
Noroozi	Autism Research	Iran	Social	990	518	.	2	.
Nuske	MA	USA	Psychology	40	20	1	2	5
O'Brien	JADD	USA	Interventions	5	5	6	2	.
O'Hearn	Autism Research	USA	Biology	26	13	.	2	1
Oerlemans	JADD	NL	Social	237	145	3	1	1
Olincy	JADD	USA	Biology	2	2	2	1	.
Ostfeld-Etzion	Autism	Israel	Psychology	80	40	.	1	1
Oswald	JADD	USA	Psychology	64	32	1	1	3, 2
Oswald	Autism Research	USA	Psychology	54	27	.	1	3
Pang	Autism Research	Canada	Biology	42	21	4	1	3
Parsons	JADD	USA	Psychology	18	8	1	1	4
Pearson	Autism Research	UK	Psychology	60	30	.	3	2
Peckett	Autism	UK	Interventions	15	5	.	3	1
Pellecchia	Autism	USA	Interventions	152	152	.	2	3
Petrina	JADD	Australia	Social	90	45	4	1	3
Poopal	MA	USA	Biology	101	58	1	2	1

Popple	JADD	USA	Interventions	18	18	.	1	1
Powell	Autism	UK	Interventions	17	17	.	2	1
Pruitt	Autism	USA	Social	166	83	.	1	3
Pugliese	JADD	USA	Psychology	64	64	1	1	1
Radley	Autism	USA	Interventions	4	2	.	1	2
Rankin	JADD	USA	Social	148	74	8	1	1, 5
Rausch	MA	Netherla nds	Biology	45	20	1	1	1
Retico	MA	Italy	Biology	152	76	4	1	1
Riches	JADD	UK	Psychology	61	30	4	1	3
Ring	Autism Research	UK	Psychology	36	18	.	1	5
Roberts	Autism	USA	Epidemiology	54512	451	.	1	3
Robinson	JADD	UK	Psychology	6	6	4	4	5
Rodgers	Autism Research	UK	Diagnosis	157	157	1	1	3
Rollins	Autism	USA	Interventions	4	4	.	1	1
Rosa	Autism	Spain	Epidemiology	128	50	1	1	1
Rosen	JADD	USA	Psychology	17	17	4	1	3
Rosso	JADD	Australia	Social	24	20	6	1	2
Rudra	MA	India	Psychology	51	25	1	3	1
Russell	Autism	UK	Psychology	859	474	.	1	1
Rutherford	Autism	UK	Epidemiology	150	150	.	1	1
Rutherford (1)	Autism	USA	Psychology	18	18	.	1	3
Rutherford (2)	Autism	USA	Psychology	17	17	.	1	1
Ryan	JADD	Ireland	Psychology	93	60	2	2	1
Rynkiewicz	MA	Poland	Social	33	33	4	2	3
Sabatos-DeVito	JADD	USA	Psychology	50	19	3	2	1, 5
Sasson	JADD	USA	Psychology	49	21	2	2	3
Scheeren	JADD	NL	Psychology	173	132	4	1	2

Schertz	JADD	USA	Psychology	143	143	2	4	3
Schuh	Autism Research	USA	Psychology	35	13	.	1	.
Schunke	Autism	Germany	Psychology	40	20	.	2	.
Schuwerk	JADD	Germany	Psychology	85	44	4	3	1, 5
Schwartzman	JADD	USA	Psychology	828	354	8	1	5
Senland	JADD	USA	Social	44	22	1	1	5
Shah	MA	UK	Diagnosis	40	20	4	1	3
Sharer	Autism Research	USA	Psychology	29	18	.	1	5
Shen	Autism Research	China	Social	1504	430	.	2	1
Shield	Autism Research	USA	Psychology	35	17	2	1	5
Shirama	JADD	Japan	Psychology	32	16	1	1	1
Simmons	JADD	USA	Interventions	50	27	6	1	1
Şimşek	Autism Research	Turkey	Social	61	35	.	1	1
Simut	JADD	Belgium	Social	30	30	.	1	2
Singer	JADD	USA	Epidemiology	1173	463	.	1	3
Skewes	JADD	Denmark	Psychology	32	14	2	1	.
Slappendel	JADD	NL	Diagnosis	198	50	1	1	1
Smith	JADD	UK	Psychology	42	21	4	5	5
Soke	JADD	USA	Epidemiology	8065	8065	4	1	3
Solomon	JADD	USA	Social	25	25	.	1	1
Song	Autism Research	China	Psychology	33	14	.	2	1
Sowden (1)	Autism Research	Germany	Psychology	105	60	.	1	1
Sowden (2)	Autism Research	UK	Psychology	36	18	.	1	3
Sparapani	JADD	USA	Social	196	196	4	1	2
Spriggs	JADD	USA	Interventions	4	4	6	1	2
Stephenson	JADD	USA	Psychology	91	42	4	1	.
Stewart	JADD	USA	Psychology	33	18	4	1	.
Suh	JADD	USA	Psychology	72	27	2	1	3

Suma	JADD	USA	Interventions	79	44	2	2	3
Sumner	JADD	UK	Psychology	95	30	1	1	2, 5
Tait	JADD	Hong Kong	Diagnosis	75	75	1	2	3
Takahashi	JADD	Japan	Psychology	44	17	4	1	5
Tavassoli	JADD	USA	Diagnosis	62	35	4	1	1
Tavassoli	Autism Research	USA	Psychology	42	21	.	1	1
Terzi	JADD	Greece	Psychology	40	20	4	1	1
Thiébaut	JADD	UK	Psychology	78	35	1	2	.
Timonen-Soivio	JADD	Finland	Epidemiology	22136	4441	1	5	1
Tirado (1)	JADD	Spain	Psychology	61	21	1	2	.
Tirado (2)	JADD	Spain	Psychology	66	22	1	2	.
Trevisan	MA	Canada	Psychology	34	17	1	1	2, 3, 5
Troyb	JADD	USA	Psychology	40	40	8	1	3
Turcotte	JADD	USA	Social	3297	3297	1	2	3
Uljarević	MA	Australia	Psychology	507	169	1	1	3
Uljarević	Autism Research	UK	Psychology	57	57	.	1	2, 5
Ure	Autism Research	Australia	Epidemiology	57	57	.	1	3
Uzefovsky	JADD	UK	Psychology	441	201	1	2	5
van Boxtel	Autism Research	Australia	Psychology	33	16	.	1	.
Van der Hallen (1)	JADD	Belgium	Psychology	54	21	4	1	1, 2
Van der Hallen (2)	JADD	Belgium	Psychology	53	26	4	1	1, 2
Vanmarcke	JADD	Belgium	Psychology	48	24	1	1	1
Vanmarcke	JADD	Belgium	Psychology	48	24	1	1	1
Virk	Autism	Denmark	Epidemiology	35059	552		1	3
Vivanti	MA	Australia	Psychology	57	36	1	2	1

Vogan	JADD	Canada	Biology	130	61	1	1	.
Vohra	JADD	USA	Epidemiology	102108	25527	8	1	1
Walsh	JADD	Canada	Psychology	46	23	1	1	3, 5
Weiss	Autism	Canada	Social	648	324	.	1	5
Wenger	MA	USA	Biology	225	75	4	1	1
Whitaker	Autism Research	UK	Psychology	32	16	.	1	2
Wicker	MA	France	Psychology	30	15	4	1	1
Wilson	Autism	UK	Diagnosis	1244	874	.	1	1
Wink	MA	USA	Interventions	31	31	4	1	.
Woodman	JADD	USA	Psychology	364	364	3	1	1, 2
Yakubova	JADD	USA	Interventions	4	4	1	1	2
Yang	MA	USA	Diagnosis	101	60	4	2	.
Yang	JADD	Australia	Diagnosis	4	4	4	1	1
Yin	MA	Taiwan	Biology	1428	335	4	1	1
Yoshimura	Autism Research	Japan	Biology	70	32	.	1	1
Young	JADD	USA	Interventions	77	77	1	1	2
Zamora	JADD	USA	Social	386	302	2	2	1
Zeedyk	JADD	USA	Social	127	127	1	1	2
Zwaigenbaum	Autism Research	Canada	Diagnosis	381	103	.	1	1
