# Supplementary material for: Selection bias on intellectual ability in autism research: a cross-sectional review and meta-analysis
Source: Mol Autism. 2019 Mar 1;10:9. doi: 10.1186/s13229-019-0260-x (PMC6397505; doi:10.1186/s13229-019-0260-x)
Supplement: Supplementary file 2 — Supplemental citation list of included studies. (DOCX 56 kb) [file 13229_2019_260_MOESM2_ESM.pdf]

Additional File 2: Citation list of included studies

Adams, R., Taylor, J., Duncan, A., & Bishop, S. (2016). Peer Victimization and Educational Outcomes in Mainstreamed Adolescents with Autism Spectrum Disorder (ASD). *J Autism Dev Disord*, *46*(11), 3557–3566. https://doi.org/10.1007/s10803-016-2893-3

Allen, M. L., & Craig, E. (2016). Brief Report: Imaginative Drawing in Children with Autism Spectrum Disorder and Learning Disabilities. *J Autism Dev Disord*, *46*(2), 704–712. https://doi.org/10.1007/s10803-015-2599-y

Alvares, G. A., Balleine, B. W., Whittle, L., & Guastella, A. J. (2016). Reduced goal-directed action control in autism spectrum disorder. *Autism Res*, *9*(12), 1285–1293. https://doi.org/10.1002/aur.1613

Anderson, A., Locke, J., Kretzmann, M., Kasari, C., & Network, A.-B. (2016). Social network analysis of children with autism spectrum disorder: Predictors of fragmentation and connectivity in elementary school classrooms. *Autism*, *20*(6), 700–709. https://doi.org/10.1177/1362361315603568

Anderson, S., & Meints, K. (2016). Brief Report: The Effects of Equine-Assisted Activities on the Social Functioning in Children and Adolescents with Autism Spectrum Disorder. *J Autism Dev Disord*, *46*(10), 3344–3352. https://doi.org/10.1007/s10803-016-2869-3

Antezana, L., Mosner, M. G., Troiani, V., & Yerys, B. E. (2016). Social-Emotional Inhibition of Return in Children with Autism Spectrum Disorder Versus Typical Development. *J Autism Dev Disord*, *46*(4), 1236–1246. https://doi.org/10.1007/s10803-015-2661-9

Ausderau, K. K., Sideris, J., Little, L. M., Furlong, M., Bulluck, J. C., & Baranek, G. T. (2016). Sensory subtypes and associated outcomes in children with autism spectrum disorders. *Autism Res*, *9*(12), 1316–1327. https://doi.org/10.1002/aur.1626

Azmitia, E. C., Saccomano, Z. T., Alzoobaee, M. F., Boldrini, M., & Whitaker-Azmitia, P. M. (2016). Persistent Angiogenesis in the Autism Brain: An Immunocytochemical Study of

Postmortem Cortex, Brainstem and Cerebellum. *J Autism Dev Disord*, *46*(4), 1307–1318. https://doi.org/10.1007/s10803-015-2672-6

Balboni, G., Tasso, A., Muratori, F., & Cubelli, R. (2016). The Vineland-II in Preschool Children with Autism Spectrum Disorders: An Item Content Category Analysis. *J Autism Dev Disord*, *46*(1), 42–52. https://doi.org/10.1007/s10803-015-2533-3

Baldwin, P. R., Curtis, K. N., Patriquin, M. A., Wolf, V., Viswanath, H., Shaw, C., … Salas, R. (2016). Identifying diagnostically-relevant resting state brain functional connectivity in the ventral posterior complex via genetic data mining in autism spectrum disorder. *Autism Res*, *9*(5), 553–562. https://doi.org/10.1002/aur.1559

Bargiela, S., Steward, R., & Mandy, W. (2016). The Experiences of Late-diagnosed Women with Autism Spectrum Conditions: An Investigation of the Female Autism Phenotype. *J Autism Dev Disord*, *46*(10), 3281–3294. https://doi.org/10.1007/s10803-016-2872-8

Barnard-Brak, L., Brewer, A., Chesnut, S., Richman, D., & Schaeffer, A. M. (2016). The sensitivity and specificity of the social communication questionnaire for autism spectrum with respect to age. *Autism Res*, *9*(8), 838–845. https://doi.org/10.1002/aur.1584

Barnevik Olsson, M., Lundstrom, S., Westerlund, J., Giacobini, M. B., Gillberg, C., & Fernell, E. (2016). Preschool to School in Autism: Neuropsychiatric Problems 8 Years After Diagnosis at 3 Years of Age. *J Autism Dev Disord*, *46*(8), 2749–2755. https://doi.org/10.1007/s10803-016-2819-0

Bearss, K., Taylor, C. A., Aman, M. G., Whittemore, R., Lecavalier, L., Miller, J., … Scahill, L. (2016). Using qualitative methods to guide scale development for anxiety in youth with autism spectrum disorder. *Autism*, *20*(6), 663–672. https://doi.org/10.1177/1362361315601012

Bedford, R., Pellicano, E., Mareschal, D., & Nardini, M. (2016). Flexible integration of visual cues in adolescents with autism spectrum disorder. *Autism Res*, *9*(2), 272–281. https://doi.org/10.1002/aur.1509

Bedford, R., Pickles, A., & Lord, C. (2016). Early gross motor skills predict the subsequent development of language in children with autism spectrum disorder. *Autism Res*, *9*(9), 993–1001. https://doi.org/10.1002/aur.1587

Begeer, S., Fink, E., van der Meijden, S., Goossens, F., & Olthof, T. (2016). Bullying-related behaviour in a mainstream high school versus a high school for autism: Self-report and peer-report. *Autism*, *20*(5), 562–571. https://doi.org/10.1177/1362361315597525

Ben-Itzchak, E., Abutbul, S., Bela, H., Shai, T., & Zachor, D. A. (2016). Understanding One's Own Emotions in Cognitively-Able Preadolescents with Autism Spectrum Disorder. *J Autism Dev Disord*, *46*(7), 2363–2371. https://doi.org/10.1007/s10803-016-2769-6

Benning, S. D., Kovac, M., Campbell, A., Miller, S., Hanna, E. K., Damiano, C. R., … Dichter, G. S. (2016). Late Positive Potential ERP Responses to Social and Nonsocial Stimuli in Youth with Autism Spectrum Disorder. *J Autism Dev Disord*, *46*(9), 3068–3077. https://doi.org/10.1007/s10803-016-2845-y

Benson, V., Castelhano, M. S., Howard, P. L., Latif, N., & Rayner, K. (2016). Looking, seeing and believing in autism: Eye movements reveal how subtle cognitive processing differences impact in the social domain. *Autism Res*, *9*(8), 879–887. https://doi.org/10.1002/aur.1580

Biggs, E. E., & Carter, E. W. (2016). Quality of Life for Transition-Age Youth with Autism or Intellectual Disability. *J Autism Dev Disord*, *46*(1), 190–204. https://doi.org/10.1007/s10803-015-2563-x

Bilaver, L. A., Cushing, L. S., & Cutler, A. T. (2016). Prevalence and Correlates of Educational Intervention Utilization Among Children with Autism Spectrum Disorder. *J Autism Dev Disord*, *46*(2), 561–571. https://doi.org/10.1007/s10803-015-2598-z

Bishop-Fitzpatrick, L., Hong, J., Smith, L. E., Makuch, R. A., Greenberg, J. S., & Mailick, M. R. (2016). Characterizing Objective Quality of Life and Normative Outcomes in Adults with Autism Spectrum Disorder: An Exploratory Latent Class Analysis. *J Autism Dev Disord*, *46*(8), 2707–2719. https://doi.org/10.1007/s10803-016-2816-3

Blackmon, K., Bluvstein, J., MacAllister, W. S., Avallone, J., Misajon, J., Hedlund, J., … Devinsky, O. (2016). Treatment Resistant Epilepsy in Autism Spectrum Disorder: Increased Risk for Females. *Autism Res*, *9*(2), 311–320. https://doi.org/10.1002/aur.1514

Blumberg, S. J., Zablotsky, B., Avila, R. M., Colpe, L. J., Pringle, B. A., & Kogan, M. D. (2016). Diagnosis lost: Differences between children who had and who currently have an autism spectrum disorder diagnosis. *Autism*, *20*(7), 783–795. https://doi.org/10.1177/1362361315607724

Bolic Baric, V., Hellberg, K., Kjellberg, A., & Hemmingsson, H. (2016). Support for learning goes beyond academic support: Voices of students with Asperger's disorder and attention deficit hyperactivity disorder. *Autism*, *20*(2), 183–195. https://doi.org/10.1177/1362361315574582

Bottema-Beutel, K., Mullins, T. S., Harvey, M. N., Gustafson, J. R., & Carter, E. W. (2016). Avoiding the 'brick wall of awkward': Perspectives of youth with autism spectrum disorder on social-focused intervention practices. *Autism*, *20*(2), 196–206. https://doi.org/10.1177/1362361315574888

Bottema-Beutel, K., & White, R. (2016). By the Book: An Analysis of Adolescents with Autism Spectrum Condition Co-constructing Fictional Narratives with Peers. *J Autism Dev Disord*, *46*(2), 361–377. https://doi.org/10.1007/s10803-015-2524-4

Bouvet, L., Mottron, L., Valdois, S., & Donnadieu, S. (2016). Auditory Stream Segregation in Autism Spectrum Disorder: Benefits and Downsides of Superior Perceptual Processes. *J Autism Dev Disord*, *46*(5), 1553–1561. https://doi.org/10.1007/s10803-013-2003-8

Brett, D., Warnell, F., McConachie, H., & Parr, J. R. (2016). Factors Affecting Age at ASD Diagnosis in UK: No Evidence that Diagnosis Age has Decreased Between 2004 and 2014. *J Autism Dev Disord*, *46*(6), 1974–1984. https://doi.org/10.1007/s10803-016-2716-6

Brewer, R., Biotti, F., Catmur, C., Press, C., Happe, F., Cook, R., & Bird, G. (2016). Can Neurotypical Individuals Read Autistic Facial Expressions? Atypical Production of Emotional Facial Expressions in Autism Spectrum Disorders. *Autism Res*, *9*(2), 262–271. https://doi.org/10.1002/aur.1508

Brian, J., Bryson, S. E., Smith, I. M., Roberts, W., Roncadin, C., Szatmari, P., & Zwaigenbaum, L. (2016). Stability and change in autism spectrum disorder diagnosis from age 3 to middle childhood in a high-risk sibling cohort. *Autism*, *20*(7), 888–892. https://doi.org/10.1177/1362361315614979

Brooks, J., Kellett, J., Seeanner, J., Jenkins, C., Buchanan, C., Kinsman, A., … Pierce, S. (2016). Training the Motor Aspects of Pre-driving Skills of Young Adults With and Without Autism Spectrum Disorder. *J Autism Dev Disord*, *46*(7), 2408–2426. https://doi.org/10.1007/s10803-016-2775-8

Brosnan, M., Johnson, H., Grawemeyer, B., Chapman, E., Antoniadou, K., & Hollinworth, M. (2016). Deficits in metacognitive monitoring in mathematics assessments in learners with autism spectrum disorder. *Autism*, *20*(4), 463–472. https://doi.org/10.1177/1362361315589477

Brosnan, M, Lewton, M., & Ashwin, C. (2016). Reasoning on the autism spectrum: A dual process theory account. *J Autism Dev Disord*, *46*(6), 2115-2125. https://doi.org/10.1007/s10803-016-2742-4

Burger-Caplan, R., Saulnier, C., Jones, W., & Klin, A. (2016). Predicting social and communicative ability in school-age children with autism spectrum disorder: A pilot study of

the Social Attribution Task, Multiple Choice. *Autism*, *20*(8), 952–962. https://doi.org/10.1177/1362361315617589

Cage, E., Bird, G., & Pellicano, E. (2016). Reputation Management in Children on the Autism Spectrum. *J Autism Dev Disord*, *46*(12), 3798–3811. https://doi.org/10.1007/s10803-016-2923-1

Cai, R. Y., & Richdale, A. L. (2016). Educational Experiences and Needs of Higher Education Students with Autism Spectrum Disorder. *J Autism Dev Disord*, *46*(1), 31–41. https://doi.org/10.1007/s10803-015-2535-1

Campione, G. C., Piazza, C., Villa, L., & Molteni, M. (2016). Three-Dimensional Kinematic Analysis of Prehension Movements in Young Children with Autism Spectrum Disorder: New Insights on Motor Impairment. *J Autism Dev Disord*, *46*(6), 1985–1999. https://doi.org/10.1007/s10803-016-2732-6

Caplan, B., Feldman, M., Eisenhower, A., & Blacher, J. (2016). Student-Teacher Relationships for Young Children with Autism Spectrum Disorder: Risk and Protective Factors. *J Autism Dev Disord*, *46*(12), 3653–3666. https://doi.org/10.1007/s10803-016-2915-1

Carmo, J. C., Duarte, E., Pinho, S., Filipe, C. N., & Marques, J. F. (2016). Preserved Proactive Interference in Autism Spectrum Disorder. *J Autism Dev Disord*, *46*(1), 53–63. https://doi.org/10.1007/s10803-015-2540-4

Cascio, C. J., Lorenzi, J., & Baranek, G. T. (2016). Self-reported Pleasantness Ratings and Examiner-Coded Defensiveness in Response to Touch in Children with ASD: Effects of Stimulus Material and Bodily Location. *J Autism Dev Disord*, *46*(5), 1528–1537. https://doi.org/10.1007/s10803-013-1961-1

Chamak, B., & Bonniau, B. (2016). Trajectories, Long-Term Outcomes and Family Experiences of 76 Adults with Autism Spectrum Disorder. *J Autism Dev Disord*, *46*(3), 1084–1095. https://doi.org/10.1007/s10803-015-2656-6

Chang, Y. C., Shih, W., & Kasari, C. (2016). Friendships in preschool children with autism spectrum disorder: What holds them back, child characteristics or teacher behavior? *Autism, 20*(1), 65–74. https://doi.org/10.1177/1362361314567761

Chen, H., Uddin, L. Q., Zhang, Y., Duan, X., & Chen, H. (2016). Atypical effective connectivity of thalamo-cortical circuits in autism spectrum disorder. *Autism Res, 9*(11), 1183–1190. https://doi.org/10.1002/aur.1614

Chen, P. J., Gau, S. S., Lee, S. H., & Chou, T. L. (2016). Differences in age-dependent neural correlates of semantic processing between youths with autism spectrum disorder and typically developing youths. *Autism Res, 9*(12), 1263–1273. https://doi.org/10.1002/aur.1616

Chen, Y. W., Bundy, A., Cordier, R., Chien, Y. L., & Einfeld, S. (2016). The Experience of Social Participation in Everyday Contexts Among Individuals with Autism Spectrum Disorders: An Experience Sampling Study. *J Autism Dev Disord*, *46*(4), 1403–1414. https://doi.org/10.1007/s10803-015-2682-4

Chiang, C. H., Chu, C. L., & Lee, T. C. (2016). Efficacy of caregiver-mediated joint engagement intervention for young children with autism spectrum disorders. *Autism*, *20*(2), 172–182. https://doi.org/10.1177/1362361315575725

Chien, H. Y., Gau, S. S., & Isaac Tseng, W. Y. (2016). Deficient visuospatial working memory functions and neural correlates of the default-mode network in adolescents with autism spectrum disorder. *Autism Res*, *9*(10), 1058–1072. https://doi.org/10.1002/aur.1607

Chiu, T. A., Anagnostou, E., Brian, J., Chau, T., & Kushki, A. (2016). Specificity of autonomic arousal to anxiety in children with autism spectrum disorder. *Autism Res*, *9*(4), 491–501. https://doi.org/10.1002/aur.1528

Cholemkery, H., Medda, J., Lempp, T., & Freitag, C. M. (2016). Classifying Autism Spectrum Disorders by ADI-R: Subtypes or Severity Gradient? *J Autism Dev Disord*, *46*(7), 2327–2339. https://doi.org/10.1007/s10803-016-2760-2

Choque Olsson, N., Rautio, D., Asztalos, J., Stoetzer, U., & Bolte, S. (2016). Social skills group training in high-functioning autism: A qualitative responder study. *Autism*, *20*(8), 995–1010. https://doi.org/10.1177/1362361315621885

Chowdhury, M., Aman, M. G., Lecavalier, L., Smith, T., Johnson, C., Swiezy, N., … Scahill, L. (2016). Factor structure and psychometric properties of the revised Home Situations Questionnaire for autism spectrum disorder: The Home Situations Questionnaire-Autism Spectrum Disorder. *Autism*, *20*(5), 528–537. https://doi.org/10.1177/1362361315593941

Christiansz, J. A., Gray, K. M., Taffe, J., & Tonge, B. J. (2016). Autism Spectrum Disorder in the DSM-5: Diagnostic Sensitivity and Specificity in Early Childhood. *J Autism Dev Disord*, *46*(6), 2054–2063. https://doi.org/10.1007/s10803-016-2734-4

Connolly, N., Anixt, J., Manning, P., Ping, I. L. D., Marsolo, K. A., & Bowers, K. (2016). Maternal metabolic risk factors for autism spectrum disorder-An analysis of electronic medical records and linked birth data. *Autism Res*, *9*(8), 829–837. https://doi.org/10.1002/aur.1586

Conson, M., Hamilton, A., De Bellis, F., Errico, D., Improta, I., Mazzarella, E., … Frolli, A. (2016). Body Constraints on Motor Simulation in Autism Spectrum Disorders. *J Autism Dev Disord*, *46*(3), 1051–1060. https://doi.org/10.1007/s10803-015-2652-x

Cooper, R. A., Plaisted-Grant, K. C., Baron-Cohen, S., & Simons, J. S. (2016). Reality Monitoring and Metamemory in Adults with Autism Spectrum Conditions. *J Autism Dev Disord*, *46*(6), 2186–2198. https://doi.org/10.1007/s10803-016-2749-x

Corbett, B. A., Key, A. P., Qualls, L., Fecteau, S., Newsom, C., Coke, C., & Yoder, P. (2016). Improvement in Social Competence Using a Randomized Trial of a Theatre Intervention for Children with Autism Spectrum Disorder. *J Autism Dev Disord*, *46*(2), 658–672. https://doi.org/10.1007/s10803-015-2600-9

Coster, W. J., Kramer, J. M., Tian, F., Dooley, M., Liljenquist, K., Kao, Y. C., & Ni, P. (2016). Evaluating the appropriateness of a new computer-administered measure of adaptive function for children and youth with autism spectrum disorders. *Autism*, *20*(1), 14–25. https://doi.org/10.1177/1362361314564473

Cox, S. M., Cox, D. J., Kofler, M. J., Moncrief, M. A., Johnson, R. J., Lambert, A. E., … Reeve, R. E. (2016). Driving Simulator Performance in Novice Drivers with Autism Spectrum Disorder: The Role of Executive Functions and Basic Motor Skills. *J Autism Dev Disord*, *46*(4), 1379–1391. https://doi.org/10.1007/s10803-015-2677-1

Crawford, H., Moss, J., Oliver, C., Elliott, N., Anderson, G. M., & McCleery, J. P. (2016). Visual preference for social stimuli in individuals with autism or neurodevelopmental disorders: an eye-tracking study. *Mol Autism*, *7*, 24. https://doi.org/10.1186/s13229-016-0084-x

Crea, K., Dissanayake, C., & Hudry, K. (2016). Proband Mental Health Difficulties and Parental Stress Predict Mental Health in Toddlers at High-Risk for Autism Spectrum Disorders. *J Autism Dev Disord*, *46*(10), 3242–3257. https://doi.org/10.1007/s10803-016-2861-y

Cummings, J. R., Lynch, F. L., Rust, K. C., Coleman, K. J., Madden, J. M., Owen-Smith, A. A., … Croen, L. A. (2016). Health Services Utilization Among Children With and Without Autism Spectrum Disorders. *J Autism Dev Disord*, *46*(3), 910–920. https://doi.org/10.1007/s10803-015-2634-z

Davids, R. C., Groen, Y., Berg, I. J., Tucha, O. M., & van Balkom, I. D. (2016). Executive Functions in Older Adults With Autism Spectrum Disorder: Objective Performance and Subjective Complaints. *J Autism Dev Disord*, *46*(9), 2859–2873. https://doi.org/10.1007/s10803-016-2831-4

de Bildt, A., Sytema, S., Meffert, H., & Bastiaansen, J. A. (2016). The Autism Diagnostic Observation Schedule, Module 4: Application of the Revised Algorithms in an Independent,

Well-Defined, Dutch Sample (n = 93). *J Autism Dev Disord*, *46*(1), 21–30. https://doi.org/10.1007/s10803-015-2532-4

Dekker, V., Nauta, M. H., Mulder, E. J., Sytema, S., & de Bildt, A. (2016). A Fresh Pair of Eyes: A Blind Observation Method for Evaluating Social Skills of Children with ASD in a Naturalistic Peer Situation in School. *Journal of Autism and Developmental Disorders*, *46*(9), 2890–2904. https://doi.org/10.1007/s10803-016-2829-y

Demopoulos, C., & Lewine, J. D. (2016). Audiometric Profiles in Autism Spectrum Disorders: Does Subclinical Hearing Loss Impact Communication? *Autism Res*, *9*(1), 107–120. https://doi.org/10.1002/aur.1495

Dempsey, J., Dempsey, A. G., Guffey, D., Minard, C. G., & Goin-Kochel, R. P. (2016). Brief Report: Further Examination of Self-Injurious Behaviors in Children and Adolescents with Autism Spectrum Disorders. *J Autism Dev Disord*, *46*(5), 1872–1879. https://doi.org/10.1007/s10803-016-2704-x

DiCriscio, A. S., Miller, S. J., Hanna, E. K., Kovac, M., Turner-Brown, L., Sasson, N. J., … Dichter, G. S. (2016). Brief Report: Cognitive Control of Social and Nonsocial Visual Attention in Autism. *J Autism Dev Disord*, *46*(8), 2797–2805. https://doi.org/10.1007/s10803-016-2804-7

Dolan, B. K., Van Hecke, A. V., Carson, A. M., Karst, J. S., Stevens, S., Schohl, K. A., … Hummel, E. (2016). Brief Report: Assessment of Intervention Effects on In Vivo Peer Interactions in Adolescents with Autism Spectrum Disorder (ASD). *J Autism Dev Disord*, *46*(6), 2251–2259. https://doi.org/10.1007/s10803-016-2738-0

Doumas, M., McKenna, R., & Murphy, B. (2016). Postural Control Deficits in Autism Spectrum Disorder: The Role of Sensory Integration. *J Autism Dev Disord*, *46*(3), 853–861. https://doi.org/10.1007/s10803-015-2621-4

Durieux, A. M., Horder, J., Mendez, M. A., Egerton, A., Williams, S. C., Wilson, C. E., … McAlonan, G. M. (2016). Cortical and subcortical glutathione levels in adults with autism spectrum disorder. *Autism Res*, *9*(4), 429–435. https://doi.org/10.1002/aur.1522

Dynia, J. M., Brock, M. E., Logan, J. A., Justice, L. M., & Kaderavek, J. N. (2016). Comparing Children with ASD and Their Peers' Growth in Print Knowledge. *J Autism Dev Disord*, *46*(7), 2490–2500. https://doi.org/10.1007/s10803-016-2790-9

Edmiston, E. K., Jones, R. M., & Corbett, B. A. (2016). Physiological Response to Social Evaluative Threat in Adolescents with Autism Spectrum Disorder. *J Autism Dev Disord*, *46*(9), 2992–3005. https://doi.org/10.1007/s10803-016-2842-1

Ekhlaspour, L., Baskaran, C., Campoverde, K. J., Sokoloff, N. C., Neumeyer, A. M., & Misra, M. (2016). Bone Density in Adolescents and Young Adults with Autism Spectrum Disorders. *J Autism Dev Disord*, *46*(11), 3387–3391. https://doi.org/10.1007/s10803-016-2871-9

Ellis Weismer, S., Haebig, E., Edwards, J., Saffran, J., & Venker, C. E. (2016). Lexical Processing in Toddlers with ASD: Does Weak Central Coherence Play a Role? *J Autism Dev Disord*, *46*(12), 3755–3769. https://doi.org/10.1007/s10803-016-2926-y

Eussen, M. L., Van Gool, A. R., Louwerse, A., Verhulst, F. C., & Greaves-Lord, K. (2016). Superior Disembedding Performance in Childhood Predicts Adolescent Severity of Repetitive Behaviors: A Seven Years Follow-Up of Individuals With Autism Spectrum Disorder. *Autism Res*, *9*(2), 282–291. https://doi.org/10.1002/aur.1510

Eversole, M., Collins, D. M., Karmarkar, A., Colton, L., Quinn, J. P., Karsbaek, R., … Hilton, C. L. (2016). Leisure Activity Enjoyment of Children with Autism Spectrum Disorders. *J Autism Dev Disord*, *46*(1), 10–20. https://doi.org/10.1007/s10803-015-2529-z

Factor, R. S., Condy, E. E., Farley, J. P., & Scarpa, A. (2016). Brief Report: Insistence on Sameness, Anxiety, and Social Motivation in Children with Autism Spectrum Disorder. *J Autism Dev Disord*, *46*(7), 2548–2554. https://doi.org/10.1007/s10803-016-2781-x

Fairthorne, J., Jacoby, P., Bourke, J., de Klerk, N., & Leonard, H. (2016). Onset of maternal psychiatric disorders after the birth of a child with autism spectrum disorder: A retrospective cohort study. *Autism*, *20*(1), 37–44. https://doi.org/10.1177/1362361314566048

Faja, S., Dawson, G., Sullivan, K., Meltzoff, A. N., Estes, A., & Bernier, R. (2016). Executive function predicts the development of play skills for verbal preschoolers with autism spectrum disorders. *Autism Res*, *9*(12), 1274–1284. https://doi.org/10.1002/aur.1608

Fernandes, L. C., Gillberg, C. I., Cederlund, M., Hagberg, B., Gillberg, C., & Billstedt, E. (2016). Aspects of Sexuality in Adolescents and Adults Diagnosed with Autism Spectrum Disorders in Childhood. *J Autism Dev Disord*, *46*(9), 3155–3165. https://doi.org/10.1007/s10803-016-2855-9

Field, C., Allen, M. L., & Lewis, C. (2016). Are Children with Autism Spectrum Disorder Initially Attuned to Object Function Rather Than Shape for Word Learning? *J Autism Dev Disord*, *46*(4), 1210–1219. https://doi.org/10.1007/s10803-015-2657-5

Findon, J., Cadman, T., Stewart, C. S., Woodhouse, E., Eklund, H., Hayward, H., … McEwen, F. S. (2016). Screening for co-occurring conditions in adults with autism spectrum disorder using the strengths and difficulties questionnaire: A pilot study. *Autism Res*, *9*(12), 1353–1363. https://doi.org/10.1002/aur.1625

Fiorentino, M., Sapone, A., Senger, S., Camhi, S. S., Kadzielski, S. M., Buie, T. M., … Fasano, A. (2016). Blood-brain barrier and intestinal epithelial barrier alterations in autism spectrum disorders. *Mol Autism*, *7*, 49. https://doi.org/10.1186/s13229-016-0110-z

Fisher, M. H., & Taylor, J. L. (2016). Let's talk about it: Peer victimization experiences as reported by adolescents with autism spectrum disorder. *Autism*, *20*(4), 402–411. https://doi.org/10.1177/1362361315585948

Fletcher-Watson, S., Petrou, A., Scott-Barrett, J., Dicks, P., Graham, C., O'Hare, A., … McConachie, H. (2016). A trial of an iPad intervention targeting social communication skills in children with autism. *Autism*, *20*(7), 771–782. https://doi.org/10.1177/1362361315605624

Floris, D. L., Barber, A. D., Nebel, M. B., Martinelli, M., Lai, M. C., Crocetti, D., … Mostofsky, S. H. (2016). Atypical lateralization of motor circuit functional connectivity in children with autism is associated with motor deficits. *Mol Autism*, *7*, 35. https://doi.org/10.1186/s13229-016-0096-6

Fombonne, E., Marcin, C., Manero, A. C., Bruno, R., Diaz, C., Villalobos, M., … Nealy, B. (2016). Prevalence of Autism Spectrum Disorders in Guanajuato, Mexico: The Leon survey. *J Autism Dev Disord*, *46*(5), 1669–1685. https://doi.org/10.1007/s10803-016-2696-6

Forbes, P. A., Pan, X., & de, C. H. A. F. (2016). Reduced Mimicry to Virtual Reality Avatars in Autism Spectrum Disorder. *J Autism Dev Disord*, *46*(12), 3788–3797. https://doi.org/10.1007/s10803-016-2930-2

Forgeot d'Arc, B., Vinckier, F., Lebreton, M., Soulieres, I., Mottron, L., & Pessiglione, M. (2016). Mimetic desire in autism spectrum disorder. *Mol Autism*, *7*, 45. https://doi.org/10.1186/s13229-016-0107-7

Foster, N. E., Ouimet, T., Tryfon, A., Doyle-Thomas, K., Anagnostou, E., & Hyde, K. L. (2016). Effects of Age and Attention on Auditory Global-Local Processing in Children with Autism Spectrum Disorder. *J Autism Dev Disord*, *46*(4), 1415–1428. https://doi.org/10.1007/s10803-015-2684-2

Fridenson-Hayo, S., Berggren, S., Lassalle, A., Tal, S., Pigat, D., Bolte, S., … Golan, O. (2016). Basic and complex emotion recognition in children with autism: cross-cultural findings. *Mol Autism*, *7*, 52. https://doi.org/10.1186/s13229-016-0113-9

Fujioka, T., Inohara, K., Okamoto, Y., Masuya, Y., Ishitobi, M., Saito, D. N., … Kosaka, H. (2016). Gazefinder as a clinical supplementary tool for discriminating between autism

spectrum disorder and typical development in male adolescents and adults. *Mol Autism*, *7*, 19. https://doi.org/10.1186/s13229-016-0083-y

Gevi, F., Zolla, L., Gabriele, S., & Persico, A. M. (2016). Urinary metabolomics of young Italian autistic children supports abnormal tryptophan and purine metabolism. *Mol Autism*, *7*, 47. https://doi.org/10.1186/s13229-016-0109-5

Gilson, C. B., & Carter, E. W. (2016). Promoting Social Interactions and Job Independence for College Students with Autism or Intellectual Disability: A Pilot Study. *J Autism Dev Disord*, *46*(11), 3583–3596. https://doi.org/10.1007/s10803-016-2894-2

Ginevra, M. C., Nota, L., & Stokes, M. A. (2016). The differential effects of Autism and Down's syndrome on sexual behavior. *Autism Res*, *9*(1), 131–140. https://doi.org/10.1002/aur.1504

Granich, J., Lin, A., Hunt, A., Wray, J., Dass, A., & Whitehouse, A. J. (2016). Obesity and associated factors in youth with an autism spectrum disorder. *Autism*, *20*(8), 916–926. https://doi.org/10.1177/1362361315616345

Green, J., Leadbitter, K., Kay, C., & Sharma, K. (2016). Autism Spectrum Disorder in Children Adopted After Early Care Breakdown. *J Autism Dev Disord*, *46*(4), 1392–1402. https://doi.org/10.1007/s10803-015-2680-6

Griffin, C., Lombardo, M. V., & Auyeung, B. (2016). Alexithymia in children with and without autism spectrum disorders. *Autism Res*, *9*(7), 773–780. https://doi.org/10.1002/aur.1569

Grove, R., Roth, I., & Hoekstra, R. A. (2016). The motivation for special interests in individuals with autism and controls: Development and validation of the special interest motivation scale. *Autism Res*, *9*(6), 677–688. https://doi.org/10.1002/aur.1560

Guy, J., Mottron, L., Berthiaume, C., & Bertone, A. (2016). The developmental trajectory of contrast sensitivity in autism spectrum disorder. *Autism Res*, *9*(8), 866–878. https://doi.org/10.1002/aur.1579

Haas, K., Costley, D., Falkmer, M., Richdale, A., Sofronoff, K., & Falkmer, T. (2016). Factors Influencing the Research Participation of Adults with Autism Spectrum Disorders. *J Autism Dev Disord*, *46*(5), 1793–1805. https://doi.org/10.1007/s10803-016-2708-6

Hagmann, C. E., Wyble, B., Shea, N., LeBlanc, M., Kates, W. R., & Russo, N. (2016). Children with Autism Detect Targets at Very Rapid Presentation Rates with Similar Accuracy as Adults. *J Autism Dev Disord*, *46*(5), 1762–1772. https://doi.org/10.1007/s10803-016-2705-9

Haigh, S. M., Minshew, N., Heeger, D. J., Dinstein, I., & Behrmann, M. (2016). Over-Responsiveness and Greater Variability in Roughness Perception in Autism. *Autism Res*, *9*(3), 393–402. https://doi.org/10.1002/aur.1505

Hanaie, R., Mohri, I., Kagitani-Shimono, K., Tachibana, M., Matsuzaki, J., Hirata, I., … Taniike, M. (2016). White matter volume in the brainstem and inferior parietal lobule is related to motor performance in children with autism spectrum disorder: A voxel-based morphometry study. *Autism Res*, *9*(9), 981–992. https://doi.org/10.1002/aur.1605

Hannah, L. A., & Stagg, S. D. (2016). Experiences of Sex Education and Sexual Awareness in Young Adults with Autism Spectrum Disorder. *J Autism Dev Disord*, *46*(12), 3678–3687. https://doi.org/10.1007/s10803-016-2906-2

Happe, F. G., Mansour, H., Barrett, P., Brown, T., Abbott, P., & Charlton, R. A. (2016). Demographic and Cognitive Profile of Individuals Seeking a Diagnosis of Autism Spectrum Disorder in Adulthood. *J Autism Dev Disord*, *46*(11), 3469–3480. https://doi.org/10.1007/s10803-016-2886-2

Hare, D. J., Gracey, C., & Wood, C. (2016). Anxiety in high-functioning autism: A pilot study of experience sampling using a mobile platform. *Autism*, *20*(6), 730–743. https://doi.org/10.1177/1362361315604817

Harriage, B., Blair, K. S., & Miltenberger, R. (2016). An Evaluation of a Parent Implemented In Situ Pedestrian Safety Skills Intervention for Individuals with Autism. *J Autism Dev Disord*, *46*(6), 2017–2027. https://doi.org/10.1007/s10803-016-2730-8

Harrop, C., Gulsrud, A., Shih, W., Hovsepyan, L., & Kasari, C. (2016). Characterizing caregiver responses to restricted and repetitive behaviors in toddlers with autism spectrum disorder. *Autism*, *20*(3), 330–342. https://doi.org/10.1177/1362361315580443

Havdahl, K. A., von Tetzchner, S., Huerta, M., Lord, C., & Bishop, S. L. (2016). Utility of the Child Behavior Checklist as a Screener for Autism Spectrum Disorder. *Autism Res*, *9*(1), 33–42. https://doi.org/10.1002/aur.1515

Hayes, S. J., Andrew, M., Elliott, D., Gowen, E., & Bennett, S. J. (2016). Low Fidelity Imitation of Atypical Biological Kinematics in Autism Spectrum Disorders Is Modulated by Self-Generated Selective Attention. *J Autism Dev Disord*, *46*(2), 502–513. https://doi.org/10.1007/s10803-015-2588-1

Hecht, P. M., Hudson, M., Connors, S. L., Tilley, M. R., Liu, X., & Beversdorf, D. Q. (2016). Maternal serotonin transporter genotype affects risk for ASD with exposure to prenatal stress. *Autism Res*, *9*(11), 1151–1160. https://doi.org/10.1002/aur.1629

Helt, M. S., & Fein, D. A. (2016). Facial Feedback and Social Input: Effects on Laughter and Enjoyment in Children with Autism Spectrum Disorders. *J Autism Dev Disord*, *46*(1), 83–94. https://doi.org/10.1007/s10803-015-2545-z

Hepburn, S. L., Blakeley-Smith, A., Wolff, B., & Reaven, J. A. (2016). Telehealth delivery of cognitive-behavioral intervention to youth with autism spectrum disorder and anxiety: A pilot study. *Autism*, *20*(2), 207–218. https://doi.org/10.1177/1362361315575164

Hewitt, A., Hall-Lande, J., Hamre, K., Esler, A. N., Punyko, J., Reichle, J., & Gulaid, A. A. (2016). Autism Spectrum Disorder (ASD) Prevalence in Somali and Non-Somali Children. *J Autism Dev Disord*, *46*(8), 2599–2608. https://doi.org/10.1007/s10803-016-2793-6

Hiller, R. M., Young, R. L., & Weber, N. (2016). Sex differences in pre-diagnosis concerns for children later diagnosed with autism spectrum disorder. *Autism*, *20*(1), 75–84. https://doi.org/10.1177/1362361314568899

Hoffmann, F., Koehne, S., Steinbeis, N., Dziobek, I., & Singer, T. (2016). Preserved Self-other Distinction During Empathy in Autism is Linked to Network Integrity of Right Supramarginal Gyrus. *J Autism Dev Disord*, *46*(2), 637–648. https://doi.org/10.1007/s10803-015-2609-0

Holmes, L. G., Himle, M. B., & Strassberg, D. S. (2016). Parental romantic expectations and parent-child sexuality communication in autism spectrum disorders. *Autism*, *20*(6), 687–699. https://doi.org/10.1177/1362361315602371

Hong, J., Bishop-Fitzpatrick, L., Smith, L. E., Greenberg, J. S., & Mailick, M. R. (2016). Factors Associated with Subjective Quality of Life of Adults with Autism Spectrum Disorder: Self-Report Versus Maternal Reports. *J Autism Dev Disord*, *46*(4), 1368–1378. https://doi.org/10.1007/s10803-015-2678-0

Howe, F. E., & Stagg, S. D. (2016). How Sensory Experiences Affect Adolescents with an Autistic Spectrum Condition within the Classroom. *J Autism Dev Disord*, *46*(5), 1656–1668. https://doi.org/10.1007/s10803-015-2693-1

Hranilovic, D., Blazevic, S., Stefulj, J., & Zill, P. (2016). DNA Methylation Analysis of HTR2A Regulatory Region in Leukocytes of Autistic Subjects. *Autism Res*, *9*(2), 204–209. https://doi.org/10.1002/aur.1519

Hundley, R. J., Shui, A., & Malow, B. A. (2016). Relationship Between Subtypes of Restricted and Repetitive Behaviors and Sleep Disturbance in Autism Spectrum Disorder. *J Autism Dev Disord*, *46*(11), 3448–3457. https://doi.org/10.1007/s10803-016-2884-4

Hyman, S. L., Stewart, P. A., Foley, J., Cain, U., Peck, R., Morris, D. D., … Smith, T. (2016). The Gluten-Free/Casein-Free Diet: A Double-Blind Challenge Trial in Children with Autism. *J Autism Dev Disord*, *46*(1), 205–220. https://doi.org/10.1007/s10803-015-2564-9

Ibrahim, G. M., Morgan, B. R., Vogan, V. M., Leung, R. C., Anagnostou, E., & Taylor, M. J. (2016). Mapping the Network of Neuropsychological Impairment in Children with Autism Spectrum Disorder: A Graph Theoretical Analysis. *J Autism Dev Disord*, *46*(12), 3770–3777. https://doi.org/10.1007/s10803-016-2929-8

Irvine, C. A., Eigsti, I. M., & Fein, D. A. (2016). Uh, Um, and Autism: Filler Disfluencies as Pragmatic Markers in Adolescents with Optimal Outcomes from Autism Spectrum Disorder. *J Autism Dev Disord*, *46*(3), 1061–1070. https://doi.org/10.1007/s10803-015-2651-y

Ishizuka, Y., & Yamamoto, J. I. (2016). Contingent imitation increases verbal interaction in children with autism spectrum disorders. *Autism*, *20*(8), 1011–1020. https://doi.org/10.1177/1362361315622856

Izuwah, D. N., Okoh, B. A., & Alikor, E. A. (2016). Clinical Pattern of Autism in Nigeria. *Autism Res*, *9*(3), 376–381. https://doi.org/10.1002/aur.1531

Jaime, M., McMahon, C. M., Davidson, B. C., Newell, L. C., Mundy, P. C., & Henderson, H. A. (2016). Brief Report: Reduced Temporal-Central EEG Alpha Coherence During Joint Attention Perception in Adolescents with Autism Spectrum Disorder. *J Autism Dev Disord*, *46*(4), 1477–1489. https://doi.org/10.1007/s10803-015-2667-3

Jashar, D. T., Brennan, L. A., Barton, M. L., & Fein, D. (2016). Cognitive and Adaptive Skills in Toddlers Who Meet Criteria for Autism in DSM-IV but not DSM-5. *J Autism Dev Disord*, *46*(12), 3667–3677. https://doi.org/10.1007/s10803-016-2901-7

Kaartinen, M., Puura, K., Himanen, S. L., Nevalainen, J., & Hietanen, J. K. (2016). Autonomic Arousal Response Habituation to Social Stimuli Among Children with Asd. *J Autism Dev Disord*, *46*(12), 3688–3699. https://doi.org/10.1007/s10803-016-2908-0

Kalb, L. G., Vasa, R. A., Ballard, E. D., Woods, S., Goldstein, M., & Wilcox, H. C. (2016). Epidemiology of Injury-Related Emergency Department Visits in the US Among Youth with Autism Spectrum Disorder. *J Autism Dev Disord*, *46*(8), 2756–2763. https://doi.org/10.1007/s10803-016-2820-7

Kana, R. K., Patriquin, M. A., Black, B. S., Channell, M. M., & Wicker, B. (2016). Altered Medial Frontal and Superior Temporal Response to Implicit Processing of Emotions in Autism. *Autism Res*, *9*(1), 55–66. https://doi.org/10.1002/aur.1496

Kanduri, C., Kantojarvi, K., Salo, P. M., Vanhala, R., Buck, G., Blancher, C., … Jarvela, I. (2016). The landscape of copy number variations in Finnish families with autism spectrum disorders. *Autism Res*, *9*(1), 9–16. https://doi.org/10.1002/aur.1502

Karhson, D. S., & Golob, E. J. (2016). Atypical sensory reactivity influences auditory attentional control in adults with autism spectrum disorders. *Autism Res*, *9*(10), 1079–1092. https://doi.org/10.1002/aur.1593

Kauschke, C., van der Beek, B., & Kamp-Becker, I. (2016). Narratives of Girls and Boys with Autism Spectrum Disorders: Gender Differences in Narrative Competence and Internal State Language. *J Autism Dev Disord*, *46*(3), 840–852. https://doi.org/10.1007/s10803-015-2620-5

Keehn, B., & Joseph, R. M. (2016a). Exploring What's Missing: What Do Target Absent Trials Reveal About Autism Search Superiority? *J Autism Dev Disord*, *46*(5), 1686–1698. https://doi.org/10.1007/s10803-016-2700-1

Keehn, B., & Joseph, R. M. (2016b). Slowed Search in the Context of Unimpaired Grouping in Autism: Evidence from Multiple Conjunction Search. *Autism Res*, *9*(3), 333–339. https://doi.org/10.1002/aur.1534

Kern Koegel, L., Ashbaugh, K., Navab, A., & Koegel, R. L. (2016). Improving Empathic Communication Skills in Adults with Autism Spectrum Disorder. *J Autism Dev Disord*, *46*(3), 921–933. https://doi.org/10.1007/s10803-015-2633-0

Kinnear, S. H., Link, B. G., Ballan, M. S., & Fischbach, R. L. (2016). Understanding the Experience of Stigma for Parents of Children with Autism Spectrum Disorder and the Role Stigma Plays in Families' Lives. *J Autism Dev Disord*, *46*(3), 942–953. https://doi.org/10.1007/s10803-015-2637-9

Kirby, A. V. (2016). Parent Expectations Mediate Outcomes for Young Adults with Autism Spectrum Disorder. *J Autism Dev Disord*, *46*(5), 1643–1655. https://doi.org/10.1007/s10803-015-2691-3

Kirkovski, M., Enticott, P. G., Hughes, M. E., Rossell, S. L., & Fitzgerald, P. B. (2016). Atypical Neural Activity in Males But Not Females with Autism Spectrum Disorder. *J Autism Dev Disord*, *46*(3), 954–963. https://doi.org/10.1007/s10803-015-2639-7

Kitzerow, J., Teufel, K., Wilker, C., & Freitag, C. M. (2016). Using the brief observation of social communication change (BOSCC) to measure autism-specific development. *Autism Res*, *9*(9), 940–950. https://doi.org/10.1002/aur.1588

Kleinhans, N. M., Reiter, M. A., Neuhaus, E., Pauley, G., Martin, N., Dager, S., & Estes, A. (2016). Subregional differences in intrinsic amygdala hyperconnectivity and hypoconnectivity in autism spectrum disorder. *Autism Res*, *9*(7), 760–772. https://doi.org/10.1002/aur.1589

Kleinhans, N. M., Richards, T., Greenson, J., Dawson, G., & Aylward, E. (2016). Altered Dynamics of the fMRI Response to Faces in Individuals with Autism. *J Autism Dev Disord*, *46*(1), 232–241. https://doi.org/10.1007/s10803-015-2565-8

Koolschijn, P. C., & Geurts, H. M. (2016). Gray Matter Characteristics in Mid and Old Aged Adults with ASD. *J Autism Dev Disord*, *46*(8), 2666–2678. https://doi.org/10.1007/s10803-016-2810-9

Kovarski, K., Thillay, A., Houy-Durand, E., Roux, S., Bidet-Caulet, A., Bonnet-Brilhault, F., & Batty, M. (2016). Brief Report: Early VEPs to Pattern-Reversal in Adolescents and Adults

with Autism. *J Autism Dev Disord*, *46*(10), 3377–3386. https://doi.org/10.1007/s10803-016-2880-8

Kranz, T. M., Kopp, M., Waltes, R., Sachse, M., Duketis, E., Jarczok, T. A., … Chiocchetti, A. G. (2016). Meta-analysis and association of two common polymorphisms of the human oxytocin receptor gene in autism spectrum disorder. *Autism Res*, *9*(10), 1036–1045. https://doi.org/10.1002/aur.1597

Kumazaki, H., Muramatsu, T., Fujisawa, T. X., Miyao, M., Matsuura, E., Okada, K., … Mimura, M. (2016). Assessment of olfactory detection thresholds in children with autism spectrum disorders using a pulse ejection system. *Mol Autism*, *7*, 6. https://doi.org/10.1186/s13229-016-0071-2

Lehnhardt, F. G., Falter, C. M., Gawronski, A., Pfeiffer, K., Tepest, R., Franklin, J., & Vogeley, K. (2016). Sex-Related Cognitive Profile in Autism Spectrum Disorders Diagnosed Late in Life: Implications for the Female Autistic Phenotype. *J Autism Dev Disord*, *46*(1), 139–154. https://doi.org/10.1007/s10803-015-2558-7

Lever, A. G., & Geurts, H. M. (2016). Age-related differences in cognition across the adult lifespan in autism spectrum disorder. *Autism Res*, *9*(6), 666–676. https://doi.org/10.1002/aur.1545

Li, P., Zhang, C., & Yi, L. (2016). Brief Report: Sensitivity of Children with Autism Spectrum Disorders to Face Appearance in Selective Trust. *J Autism Dev Disord*, *46*(7), 2520–2525. https://doi.org/10.1007/s10803-016-2761-1

Libero, L. E., Reid, M. A., White, D. M., Salibi, N., Lahti, A. C., & Kana, R. K. (2016). Biochemistry of the cingulate cortex in autism: An MR spectroscopy study. *Autism Res*, *9*(6), 643–657. https://doi.org/10.1002/aur.1562

Liew, Z., Ritz, B., Virk, J., & Olsen, J. (2016). Maternal use of acetaminophen during pregnancy and risk of autism spectrum disorders in childhood: A Danish national birth cohort study. *Autism Res*, *9*(9), 951–958. https://doi.org/10.1002/aur.1591

Lim, C. K., Essa, M. M., de Paula Martins, R., Lovejoy, D. B., Bilgin, A. A., Waly, M. I., … Guillemin, G. J. (2016). Altered kynurenine pathway metabolism in autism: Implication for immune-induced glutamatergic activity. *Autism Res*, *9*(6), 621–631. https://doi.org/10.1002/aur.1565

Liu, X., Shimada, T., Otowa, T., Wu, Y. Y., Kawamura, Y., Tochigi, M., … Gau, S. S. (2016). Genome-wide Association Study of Autism Spectrum Disorder in the East Asian Populations. *Autism Res*, *9*(3), 340–349. https://doi.org/10.1002/aur.1536

Locke, J., Shih, W., Kretzmann, M., & Kasari, C. (2016). Examining playground engagement between elementary school children with and without autism spectrum disorder. *Autism*, *20*(6), 653–662. https://doi.org/10.1177/1362361315599468

Luo, S. X., Shinall, J. A., Peterson, B. S., & Gerber, A. J. (2016). Semantic mapping reveals distinct patterns in descriptions of social relations in adults with autism spectrum disorder. *Autism Res*, *9*(8), 846–853. https://doi.org/10.1002/aur.1581

Ma, N. S., Thompson, C., & Weston, S. (2016). Brief Report: Scurvy as a Manifestation of Food Selectivity in Children with Autism. *J Autism Dev Disord*, *46*(4), 1464–1470. https://doi.org/10.1007/s10803-015-2660-x

Macizo, P., Soriano, M. F., & Paredes, N. (2016). Phonological and Visuospatial Working Memory in Autism Spectrum Disorders. *J Autism Dev Disord*, *46*(9), 2956–2967. https://doi.org/10.1007/s10803-016-2835-0

Mackie, M. A., & Fan, J. (2016). Reduced Efficiency and Capacity of Cognitive Control in Autism Spectrum Disorder. *Autism Res*, *9*(3), 403–414. https://doi.org/10.1002/aur.1517

MacMullin, J. A., Lunsky, Y., & Weiss, J. A. (2016). Plugged in: Electronics use in youth and young adults with autism spectrum disorder. *Autism*, *20*(1), 45–54. https://doi.org/10.1177/1362361314566047

Mandy, W., Murin, M., Baykaner, O., Staunton, S., Cobb, R., Hellriegel, J., … Skuse, D. (2016). Easing the transition to secondary education for children with autism spectrum disorder: An evaluation of the Systemic Transition in Education Programme for Autism Spectrum Disorder (STEP-ASD). *Autism*, *20*(5), 580–590. https://doi.org/10.1177/1362361315598892

Mari-Bauset, S., Llopis-Gonzalez, A., Zazpe, I., Mari-Sanchis, A., & Suarez-Varela, M. M. (2016). Nutritional Impact of a Gluten-Free Casein-Free Diet in Children with Autism Spectrum Disorder. *J Autism Dev Disord*, *46*(2), 673–684. https://doi.org/10.1007/s10803-015-2582-7

Marini, A., Ferretti, F., Chiera, A., Magni, R., Adornetti, I., Nicchiarelli, S., … Valeri, G. (2016). Brief Report: Self-Based and Mechanical-Based Future Thinking in Children with Autism Spectrum Disorder. *J Autism Dev Disord*, *46*(10), 3353–3360. https://doi.org/10.1007/s10803-016-2867-5

Marler, S., Ferguson, B. J., Lee, E. B., Peters, B., Williams, K. C., McDonnell, E., … Veenstra-VanderWeele, J. (2016). Brief Report: Whole Blood Serotonin Levels and Gastrointestinal Symptoms in Autism Spectrum Disorder. *J Autism Dev Disord*, *46*(3), 1124–1130. https://doi.org/10.1007/s10803-015-2646-8

Mazurek, M. O., & Sohl, K. (2016). Sleep and Behavioral Problems in Children with Autism Spectrum Disorder. *J Autism Dev Disord*, *46*(6), 1906–1915. https://doi.org/10.1007/s10803-016-2723-7

McCormick, C., Hepburn, S., Young, G. S., & Rogers, S. J. (2016). Sensory symptoms in children with autism spectrum disorder, other developmental disorders and typical development: A longitudinal study. *Autism*, *20*(5), 572–579. https://doi.org/10.1177/1362361315599755

McCoy, S. M., Jakicic, J. M., & Gibbs, B. B. (2016). Comparison of Obesity, Physical Activity, and Sedentary Behaviors Between Adolescents With Autism Spectrum Disorders and Without. *J Autism Dev Disord*, *46*(7), 2317–2326. https://doi.org/10.1007/s10803-016-2762-0

McVey, A. J., Dolan, B. K., Willar, K. S., Pleiss, S., Karst, J. S., Casnar, C. L., … Van Hecke, A. V. (2016). A Replication and Extension of the PEERS(R) for Young Adults Social Skills Intervention: Examining Effects on Social Skills and Social Anxiety in Young Adults with Autism Spectrum Disorder. *J Autism Dev Disord*, *46*(12), 3739–3754. https://doi.org/10.1007/s10803-016-2911-5

Mhatre, D., Bapat, D., & Udani, V. (2016). Long-Term Outcomes in Children Diagnosed with Autism Spectrum Disorders in India. *J Autism Dev Disord*, *46*(3), 760–772. https://doi.org/10.1007/s10803-015-2613-4

Minshawi, N. F., Wink, L. K., Shaffer, R., Plawecki, M. H., Posey, D. J., Liu, H., … Erickson, C. A. (2016). Alexithymia in Adolescents with Autism Spectrum Disorder: Its Relationship to Internalising Difficulties, Sensory Modulation and Social Cognition. *Mol Autism*, *7*, 2. https://doi.org/10.1186/s13229-015-0062-8

Moore, D. J., Reidy, J., & Heavey, L. (2016). Attentional allocation of autism spectrum disorder individuals: Searching for a Face-in-the-Crowd. *Autism*, *20*(2), 163–171. https://doi.org/10.1177/1362361315573637

Morett, L. M., O'Hearn, K., Luna, B., & Ghuman, A. S. (2016). Altered Gesture and Speech Production in ASD Detract from In-Person Communicative Quality. *J Autism Dev Disord*, *46*(3), 998–1012. https://doi.org/10.1007/s10803-015-2645-9

Mouga, S., Cafe, C., Almeida, J., Marques, C., Duque, F., & Oliveira, G. (2016). Intellectual Profiles in the Autism Spectrum and Other Neurodevelopmental Disorders. *J Autism Dev Disord*, *46*(9), 2940–2955. https://doi.org/10.1007/s10803-016-2838-x

Muller, N., Baumeister, S., Dziobek, I., Banaschewski, T., & Poustka, L. (2016). Validation of the Movie for the Assessment of Social Cognition in Adolescents with ASD: Fixation Duration and Pupil Dilation as Predictors of Performance. *J Autism Dev Disord*, *46*(9), 2831–2844. https://doi.org/10.1007/s10803-016-2828-z

Murdaugh, D. L., Deshpande, H. D., & Kana, R. K. (2016). The Impact of Reading Intervention on Brain Responses Underlying Language in Children With Autism. *Autism Res*, *9*(1), 141–154. https://doi.org/10.1002/aur.1503

Mutluer, T., Karakoc Demirkaya, S., & Abali, O. (2016). Assessment of sleep problems and related risk factors observed in Turkish children with Autism spectrum disorders. *Autism Res*, *9*(5), 536–542. https://doi.org/10.1002/aur.1542

Nader, A. M., Courchesne, V., Dawson, M., & Soulieres, I. (2016). Does WISC-IV Underestimate the Intelligence of Autistic Children? *J Autism Dev Disord*, *46*(5), 1582–1589. https://doi.org/10.1007/s10803-014-2270-z

Neuhaus, E., Bernier, R. A., & Beauchaine, T. P. (2016). Children with Autism Show Altered Autonomic Adaptation to Novel and Familiar Social Partners. *Autism Res*, *9*(5), 579–591. https://doi.org/10.1002/aur.1543

Newbutt, N., Sung, C., Kuo, H. J., Leahy, M. J., Lin, C. C., & Tong, B. (2016). Brief Report: A Pilot Study of the Use of a Virtual Reality Headset in Autism Populations. *J Autism Dev Disord*, *46*(9), 3166–3176. https://doi.org/10.1007/s10803-016-2830-5

Nguyen, L. S., Lepleux, M., Makhlouf, M., Martin, C., Fregeac, J., Siquier-Pernet, K., … Colleaux, L. (2016). Profiling olfactory stem cells from living patients identifies miRNAs relevant for autism pathophysiology. *Mol Autism*, *7*, 1. https://doi.org/10.1186/s13229-015-0064-6

Noroozi, R., Taheri, M., Movafagh, A., Mirfakhraie, R., Solgi, G., Sayad, A., … Darvish, H. (2016). Glutamate receptor, metabotropic 7 (GRM7) gene variations and susceptibility to autism: A case-control study. *Autism Res*, *9*(11), 1161–1168. https://doi.org/10.1002/aur.1640

Nuske, H. J., Vivanti, G., & Dissanayake, C. (2016). Others' emotions teach, but not in autism: an eye-tracking pupillometry study. *Mol Autism*, *7*(1), 36. https://doi.org/10.1186/s13229-016-0098-4

O'Brien, A., Schlosser, R. W., Shane, H. C., Abramson, J., Allen, A. A., Flynn, S., … Dimery, K. (2016). Brief Report: Just-in-Time Visual Supports to Children with Autism via the Apple Watch:(R) A Pilot Feasibility Study. *J Autism Dev Disord*, *46*(12), 3818–3823. https://doi.org/10.1007/s10803-016-2891-5

Oerlemans, A. M., Hartman, C. A., Franke, B., Buitelaar, J. K., & Rommelse, N. N. (2016). Does the cognitive architecture of simplex and multiplex ASD families differ? *J Autism Dev Disord*, *46*(2), 489–501. https://doi.org/10.1007/s10803-015-2572-9

O'Hearn, K., Velanova, K., Lynn, A., Wright, C., Hallquist, M., Minshew, N., & Luna, B. (2016). Abnormalities in brain systems supporting individuation and enumeration in autism. *Autism Res*, *9*(1), 82–96. https://doi.org/10.1002/aur.1498

Olincy, A., Blakeley-Smith, A., Johnson, L., Kem, W. R., & Freedman, R. (2016). Brief Report: Initial Trial of Alpha7-Nicotinic Receptor Stimulation in Two Adult Patients with Autism Spectrum Disorder. *J Autism Dev Disord*, *46*(12), 3812–3817. https://doi.org/10.1007/s10803-016-2890-6

Ostfeld-Etzion, S., Feldman, R., Hirschler-Guttenberg, Y., Laor, N., & Golan, O. (2016). Self-regulated compliance in preschoolers with autism spectrum disorder: The role of temperament and parental disciplinary style. *Autism*, *20*(7), 868–878. https://doi.org/10.1177/1362361315615467

Oswald, T. M., Beck, J. S., Iosif, A. M., McCauley, J. B., Gilhooly, L. J., Matter, J. C., & Solomon, M. (2016). Clinical and Cognitive Characteristics Associated with Mathematics Problem Solving in Adolescents with Autism Spectrum Disorder. *Autism Res*, *9*(4), 480–490. https://doi.org/10.1002/aur.1524

Oswald, T. M., Winter-Messiers, M. A., Gibson, B., Schmidt, A. M., Herr, C. M., & Solomon, M. (2016). Sex Differences in Internalizing Problems During Adolescence in Autism Spectrum Disorder. *J Autism Dev Disord*, *46*(2), 624–636. https://doi.org/10.1007/s10803-015-2608-1

Pang, E. W., Valica, T., MacDonald, M. J., Taylor, M. J., Brian, J., Lerch, J. P., & Anagnostou, E. (2016). Abnormal Brain Dynamics Underlie Speech Production in Children with Autism Spectrum Disorder. *Autism Res*, *9*(2), 249–261. https://doi.org/10.1002/aur.1526

Parsons, T. D., & Carlew, A. R. (2016). Bimodal Virtual Reality Stroop for Assessing Distractor Inhibition in Autism Spectrum Disorders. *J Autism Dev Disord*, *46*(4), 1255–1267. https://doi.org/10.1007/s10803-015-2663-7

Pearson, A., Marsh, L., Ropar, D., & Hamilton, A. (2016). Cognitive Mechanisms underlying visual perspective taking in typical and ASC children. *Autism Res*, *9*(1), 121–130. https://doi.org/10.1002/aur.1501

Peckett, H., MacCallum, F., & Knibbs, J. (2016). Maternal experience of Lego Therapy in families with children with autism spectrum conditions: What is the impact on family relationships? *Autism*, *20*(7), 879–887. https://doi.org/10.1177/1362361315621054

Pellecchia, M., Connell, J. E., Kerns, C. M., Xie, M., Marcus, S. C., & Mandell, D. S. (2016). Child characteristics associated with outcome for children with autism in a school-based behavioral intervention. *Autism*, *20*(3), 321–329. https://doi.org/10.1177/1362361315577518

Petrina, N., Carter, M., Stephenson, J., & Sweller, N. (2016). Perceived Friendship Quality of Children with Autism Spectrum Disorder as Compared to their Peers in Mixed and Non-

mixed Dyads. *J Autism Dev Disord*, *46*(4), 1334–1343. https://doi.org/10.1007/s10803-015-2673-5

Poopal, A. C., Schroeder, L. M., Horn, P. S., Bassell, G. J., & Gross, C. (2016). Increased expression of the PI3K catalytic subunit p110δ underlies elevated S6 phosphorylation and protein synthesis in an individual with autism from a multiplex family. *Molecular Autism*, *7*, 3. https://doi.org/10.1186/s13229-015-0066-4

Popple, B., Wall, C., Flink, L., Powell, K., Discepolo, K., Keck, D., … Shic, F. (2016). Brief Report: Remotely Delivered Video Modeling for Improving Oral Hygiene in Children with ASD: A Pilot Study. *J Autism Dev Disord*, *46*(8), 2791–2796. https://doi.org/10.1007/s10803-016-2795-4

Powell, G., Wass, S. V., Erichsen, J. T., & Leekam, S. R. (2016). First evidence of the feasibility of gaze-contingent attention training for school children with autism. *Autism*, *20*(8), 927–937. https://doi.org/10.1177/1362361315617880

Pruitt, M. M., Willis, K., Timmons, L., & Ekas, N. V. (2016). The impact of maternal, child, and family characteristics on the daily well-being and parenting experiences of mothers of children with autism spectrum disorder. *Autism*, *20*(8), 973–985. https://doi.org/10.1177/1362361315620409

Pugliese, C. E., Anthony, L. G., Strang, J. F., Dudley, K., Wallace, G. L., Naiman, D. Q., & Kenworthy, L. (2016). Longitudinal Examination of Adaptive Behavior in Autism Spectrum Disorders: Influence of Executive Function. *J Autism Dev Disord*, *46*(2), 467–477. https://doi.org/10.1007/s10803-015-2584-5

Radley, K. C., Hanglein, J., & Arak, M. (2016). School-based social skills training for preschool-age children with autism spectrum disorder. *Autism*, *20*(8), 938–951. https://doi.org/10.1177/1362361315617361

Rankin, J. A., Weber, R. J., Kang, E., & Lerner, M. D. (2016). Parent- and Self-Reported Social Skills Importance in Autism Spectrum Disorder. *J Autism Dev Disord*, *46*(1), 273–286. https://doi.org/10.1007/s10803-015-2574-7

Rausch, A., Zhang, W., Haak, K. V., Mennes, M., Hermans, E. J., van Oort, E., … Groen, W. B. (2016). Altered functional connectivity of the amygdaloid input nuclei in adolescents and young adults with autism spectrum disorder: a resting state fMRI study. *Mol Autism*, *7*, 13. https://doi.org/10.1186/s13229-015-0060-x

Retico, A., Giuliano, A., Tancredi, R., Cosenza, A., Apicella, F., Narzisi, A., … Calderoni, S. (2016). The effect of gender on the neuroanatomy of children with autism spectrum disorders: a support vector machine case-control study. *Mol Autism*, *7*, 5. https://doi.org/10.1186/s13229-015-0067-3

Riches, N. G., Loucas, T., Baird, G., Charman, T., & Simonoff, E. (2016). Elephants in Pyjamas: Testing the Weak Central Coherence Account of Autism Spectrum Disorders Using a Syntactic Disambiguation Task. *J Autism Dev Disord*, *46*(1), 155–163. https://doi.org/10.1007/s10803-015-2560-0

Ring, M., Gaigg, S. B., & Bowler, D. M. (2016). Relational Memory Processes in Adults with Autism Spectrum Disorder. *Autism Res*, *9*(1), 97–106. https://doi.org/10.1002/aur.1493

Roberts, A. L., Lyall, K., Rich-Edwards, J. W., Ascherio, A., & Weisskopf, M. G. (2016). Maternal exposure to intimate partner abuse before birth is associated with autism spectrum disorder in offspring. *Autism*, *20*(1), 26–36. https://doi.org/10.1177/1362361314566049

Robinson, A., & Elliott, R. (2016). Brief Report: An Observational Measure of Empathy for Autism Spectrum: A Preliminary Study of the Development and Reliability of the Client Emotional Processing Scale. *J Autism Dev Disord*, *46*(6), 2240–2250. https://doi.org/10.1007/s10803-016-2727-3

Rodgers, J., Wigham, S., McConachie, H., Freeston, M., Honey, E., & Parr, J. R. (2016). Development of the anxiety scale for children with autism spectrum disorder (ASC-ASD). *Autism Res*, *9*(11), 1205–1215. https://doi.org/10.1002/aur.1603

Rollins, P. R., Campbell, M., Hoffman, R. T., & Self, K. (2016). A community-based early intervention program for toddlers with autism spectrum disorders. *Autism*, *20*(2), 219–232. https://doi.org/10.1177/1362361315577217

Rosa, M., Puig, O., Lazaro, L., & Calvo, R. (2016). Socioeconomic status and intelligence quotient as predictors of psychiatric disorders in children and adolescents with high-functioning autism spectrum disorder and in their siblings. *Autism*, *20*(8), 963–972. https://doi.org/10.1177/1362361315617881

Rosen, T. E., & Lerner, M. D. (2016). Externalizing and Internalizing Symptoms Moderate Longitudinal Patterns of Facial Emotion Recognition in Autism Spectrum Disorder. *J Autism Dev Disord*, *46*(8), 2621–2634. https://doi.org/10.1007/s10803-016-2800-y

Rosso, E. G. (2016). Brief Report: Coaching Adolescents with Autism Spectrum Disorder in a School-Based Multi-Sport Program. *J Autism Dev Disord*, *46*(7), 2526–2531. https://doi.org/10.1007/s10803-016-2759-8

Rudra, A., Ram, J. R., Loucas, T., Belmonte, M. K., & Chakrabarti, B. (2016). Bengali translation and characterisation of four cognitive and trait measures for autism spectrum conditions in India. *Mol Autism*, *7*, 50. https://doi.org/10.1186/s13229-016-0111-y

Russell, A. J., Murphy, C. M., Wilson, E., Gillan, N., Brown, C., Robertson, D. M., … Murphy, D. G. (2016). The mental health of individuals referred for assessment of autism spectrum disorder in adulthood: A clinic report. *Autism*, *20*(5), 623–627. https://doi.org/10.1177/1362361315604271

Rutherford, M. D., & Subiaul, F. (2016). Children with autism spectrum disorder have an exceptional explanatory drive. *Autism*, *20*(6), 744–753. https://doi.org/10.1177/1362361315605973

Rutherford, M., McKenzie, K., Johnson, T., Catchpole, C., O'Hare, A., McClure, I., … Murray, A. (2016). Gender ratio in a clinical population sample, age of diagnosis and duration of assessment in children and adults with autism spectrum disorder. *Autism*, *20*(5), 628–634. https://doi.org/10.1177/1362361315617879

Ryan, C., Stafford, M., & King, R. J. (2016). Brief Report: Seeing the Man in the Moon: Do Children with Autism Perceive Pareidolic Faces? A Pilot Study. *J Autism Dev Disord*, *46*(12), 3838–3843. https://doi.org/10.1007/s10803-016-2927-x

Rynkiewicz, A., Schuller, B., Marchi, E., Piana, S., Camurri, A., Lassalle, A., & Baron-Cohen, S. (2016). An investigation of the 'female camouflage effect' in autism using a computerized ADOS-2 and a test of sex/gender differences. *Mol Autism*, *7*, 10. https://doi.org/10.1186/s13229-016-0073-0

Sabatos-DeVito, M., Schipul, S. E., Bulluck, J. C., Belger, A., & Baranek, G. T. (2016). Eye Tracking Reveals Impaired Attentional Disengagement Associated with Sensory Response Patterns in Children with Autism. *J Autism Dev Disord*, *46*(4), 1319–1333. https://doi.org/10.1007/s10803-015-2681-5

Sasson, N. J., Shasteen, J. R., & Pinkham, A. E. (2016). Brief Report: Reduced Prioritization of Facial Threat in Adults with Autism. *J Autism Dev Disord*, *46*(4), 1471–1476. https://doi.org/10.1007/s10803-015-2664-6

Scheeren, A. M., Banerjee, R., Koot, H. M., & Begeer, S. (2016). Self-Presentation and the Role of Perspective Taking and Social Motivation in Autism Spectrum Disorder. *J Autism Dev Disord*, *46*(2), 649–657. https://doi.org/10.1007/s10803-015-2610-7

Schertz, H. H., Odom, S. L., Baggett, K. M., & Sideris, J. H. (2016). Parent-Reported Repetitive Behavior in Toddlers on the Autism Spectrum. *J Autism Dev Disord*, *46*(10), 3308–3316. https://doi.org/10.1007/s10803-016-2870-x

Schuh, J. M., Eigsti, I. M., & Mirman, D. (2016). Discourse comprehension in autism spectrum disorder: Effects of working memory load and common ground. *Autism Res*, *9*(12), 1340–1352. https://doi.org/10.1002/aur.1632

Schunke, O., Schottle, D., Vettorazzi, E., Brandt, V., Kahl, U., Baumer, T., … Munchau, A. (2016). Mirror me: Imitative responses in adults with autism. *Autism*, *20*(2), 134–144. https://doi.org/10.1177/1362361315571757

Schuwerk, T., Sodian, B., & Paulus, M. (2016). Cognitive Mechanisms Underlying Action Prediction in Children and Adults with Autism Spectrum Condition. *J Autism Dev Disord*, *46*(12), 3623–3639. https://doi.org/10.1007/s10803-016-2899-x

Schwartzman, B. C., Wood, J. J., & Kapp, S. K. (2016). Can the Five Factor Model of Personality Account for the Variability of Autism Symptom Expression? Multivariate Approaches to Behavioral Phenotyping in Adult Autism Spectrum Disorder. *J Autism Dev Disord*, *46*(1), 253–272. https://doi.org/10.1007/s10803-015-2571-x

Senland, A. K., & Higgins-D'Alessandro, A. (2016). Sociomoral Reasoning, Empathy, and Meeting Developmental Tasks During the Transition to Adulthood in Autism Spectrum Disorder. *J Autism Dev Disord*, *46*(9), 3090–3105. https://doi.org/10.1007/s10803-016-2849-7

Shah, P., Catmur, C., & Bird, G. (2016). Emotional decision-making in autism spectrum disorder: the roles of interoception and alexithymia. *Mol Autism*, *7*, 43. https://doi.org/10.1186/s13229-016-0104-x

Sharer, E. A., Mostofsky, S. H., Pascual-Leone, A., & Oberman, L. M. (2016). Isolating Visual and Proprioceptive Components of Motor Sequence Learning in ASD. *Autism Res*, *9*(5), 563–569. https://doi.org/10.1002/aur.1537

Shen, Y., Xun, G., Guo, H., He, Y., Ou, J., Dong, H., … Zhao, J. (2016). Association and gene-gene interactions study of reelin signaling pathway related genes with autism in the Han Chinese population. *Autism Res*, *9*(4), 436–442. https://doi.org/10.1002/aur.1540

Shield, A., Pyers, J., Martin, A., & Tager-Flusberg, H. (2016). Relations between language and cognition in native-signing children with autism spectrum disorder. *Autism Res*, *9*(12), 1304–1315. https://doi.org/10.1002/aur.1621

Shirama, A., Kanai, C., Kato, N., & Kashino, M. (2016). Ocular Fixation Abnormality in Patients with Autism Spectrum Disorder. *J Autism Dev Disord*, *46*(5), 1613–1622. https://doi.org/10.1007/s10803-015-2688-y

Simmons, E. S., Paul, R., & Shic, F. (2016). Brief Report: A Mobile Application to Treat Prosodic Deficits in Autism Spectrum Disorder and Other Communication Impairments: A Pilot Study. *J Autism Dev Disord*, *46*(1), 320–327. https://doi.org/10.1007/s10803-015-2573-8

Simsek, S., Cetin, I., Cim, A., & Kaya, S. (2016). Elevated levels of tissue plasminogen activator and E-selectin in male children with autism spectrum disorder. *Autism Res*, *9*(12), 1241–1247. https://doi.org/10.1002/aur.1638

Simut, R. E., Vanderfaeillie, J., Peca, A., Van de Perre, G., & Vanderborght, B. (2016). Children with Autism Spectrum Disorders Make a Fruit Salad with Probo, the Social Robot: An Interaction Study. *J Autism Dev Disord*, *46*(1), 113–126. https://doi.org/10.1007/s10803-015-2556-9

Singer, A. B., Windham, G. C., Croen, L. A., Daniels, J. L., Lee, B. K., Qian, Y., … Burstyn, I. (2016). Maternal Exposure to Occupational Asthmagens During Pregnancy and Autism

Spectrum Disorder in the Study to Explore Early Development. *J Autism Dev Disord*, *46*(11), 3458–3468. https://doi.org/10.1007/s10803-016-2882-6

Skewes, J. C., & Gebauer, L. (2016). Brief Report: Suboptimal Auditory Localization in Autism Spectrum Disorder: Support for the Bayesian Account of Sensory Symptoms. *J Autism Dev Disord*, *46*(7), 2539–2547. https://doi.org/10.1007/s10803-016-2774-9

Slappendel, G., Mandy, W., van der Ende, J., Verhulst, F. C., van der Sijde, A., Duvekot, J., … Greaves-Lord, K. (2016). Utility of the 3Di Short Version for the Diagnostic Assessment of Autism Spectrum Disorder and Compatibility with DSM-5. *J Autism Dev Disord*, *46*(5), 1834–1846. https://doi.org/10.1007/s10803-016-2713-9

Smith, A. D., Kenny, L., Rudnicka, A., Briscoe, J., & Pellicano, E. (2016). Drawing Firmer Conclusions: Autistic Children Show No Evidence of a Local Processing Bias in a Controlled Copying Task. *J Autism Dev Disord*, *46*(11), 3481–3492. https://doi.org/10.1007/s10803-016-2889-z

Soke, G. N., Rosenberg, S. A., Hamman, R. F., Fingerlin, T., Robinson, C., Carpenter, L., … DiGuiseppi, C. (2016). Brief Report: Prevalence of Self-injurious Behaviors among Children with Autism Spectrum Disorder-A Population-Based Study. *J Autism Dev Disord*, *46*(11), 3607–3614. https://doi.org/10.1007/s10803-016-2879-1

Solomon, O., Heritage, J., Yin, L., Maynard, D. W., & Bauman, M. L. (2016). 'What Brings Him Here Today?': Medical Problem Presentation Involving Children with Autism Spectrum Disorders and Typically Developing Children. *J Autism Dev Disord*, *46*(2), 378–393. https://doi.org/10.1007/s10803-015-2550-2

Song, Y., Hakoda, Y., & Sang, B. (2016). A selective impairment in extracting fearful information from another's eyes in Autism. *Autism Res*, *9*(9), 1002–1011. https://doi.org/10.1002/aur.1583

Sowden, S., Koehne, S., Catmur, C., Dziobek, I., & Bird, G. (2016). Intact Automatic Imitation and Typical Spatial Compatibility in Autism Spectrum Disorder: Challenging the Broken Mirror Theory. *Autism Res*, *9*(2), 292–300. https://doi.org/10.1002/aur.1511

Sparapani, N., Morgan, L., Reinhardt, V. P., Schatschneider, C., & Wetherby, A. M. (2016). Evaluation of Classroom Active Engagement in Elementary Students with Autism Spectrum Disorder. *J Autism Dev Disord*, *46*(3), 782–796. https://doi.org/10.1007/s10803-015-2615-2

Spriggs, A. D., Gast, D. L., & Knight, V. F. (2016). Video Modeling and Observational Learning to Teach Gaming Access to Students with ASD. *J Autism Dev Disord*, *46*(9), 2845–2858. https://doi.org/10.1007/s10803-016-2824-3

Stephenson, K. G., Quintin, E. M., & South, M. (2016). Age-Related Differences in Response to Music-Evoked Emotion Among Children and Adolescents with Autism Spectrum Disorders. *J Autism Dev Disord*, *46*(4), 1142–1151. https://doi.org/10.1007/s10803-015-2624-1

Stewart, C. R., Sanchez, S. S., Grenesko, E. L., Brown, C. M., Chen, C. P., Keehn, B., … Muller, R. A. (2016). Sensory Symptoms and Processing of Nonverbal Auditory and Visual Stimuli in Children with Autism Spectrum Disorder. *J Autism Dev Disord*, *46*(5), 1590–1601. https://doi.org/10.1007/s10803-015-2367-z

Suh, J., Orinstein, A., Barton, M., Chen, C. M., Eigsti, I. M., Ramirez-Esparza, N., & Fein, D. (2016). Ratings of Broader Autism Phenotype and Personality Traits in Optimal Outcomes from Autism Spectrum Disorder. *J Autism Dev Disord*, *46*(11), 3505–3518. https://doi.org/10.1007/s10803-016-2868-4

Suma, K., Adamson, L. B., Bakeman, R., Robins, D. L., & Abrams, D. N. (2016). After Early Autism Diagnosis: Changes in Intervention and Parent-Child Interaction. *J Autism Dev Disord*, *46*(8), 2720–2733. https://doi.org/10.1007/s10803-016-2808-3

Sumner, E., Leonard, H. C., & Hill, E. L. (2016). Overlapping Phenotypes in Autism Spectrum Disorder and Developmental Coordination Disorder: A Cross-Syndrome Comparison of

Motor and Social Skills. *J Autism Dev Disord*, *46*(8), 2609–2620. https://doi.org/10.1007/s10803-016-2794-5

Tait, K., Fung, F., Hu, A., Sweller, N., & Wang, W. (2016). Understanding Hong Kong Chinese Families' Experiences of an Autism/ASD Diagnosis. *J Autism Dev Disord*, *46*(4), 1164–1183. https://doi.org/10.1007/s10803-015-2650-z

Takahashi, H., Komatsu, S., Nakahachi, T., Ogino, K., & Kamio, Y. (2016). Relationship of the Acoustic Startle Response and Its Modulation to Emotional and Behavioral Problems in Typical Development Children and Those with Autism Spectrum Disorders. *J Autism Dev Disord*, *46*(2), 534–543. https://doi.org/10.1007/s10803-015-2593-4

Tavassoli, T., Bellesheim, K., Siper, P. M., Wang, A. T., Halpern, D., Gorenstein, M., … Buxbaum, J. D. (2016). Measuring Sensory Reactivity in Autism Spectrum Disorder: Application and Simplification of a Clinician-Administered Sensory Observation Scale. *J Autism Dev Disord*, *46*(1), 287–293. https://doi.org/10.1007/s10803-015-2578-3

Tavassoli, T., Bellesheim, K., Tommerdahl, M., Holden, J. M., Kolevzon, A., & Buxbaum, J. D. (2016). Altered tactile processing in children with autism spectrum disorder. *Autism Res*, *9*(6), 616–620. https://doi.org/10.1002/aur.1563

Terzi, A., Marinis, T., & Francis, K. (2016). The Interface of Syntax with Pragmatics and Prosody in Children with Autism Spectrum Disorders. *J Autism Dev Disord*, *46*(8), 2692–2706. https://doi.org/10.1007/s10803-016-2811-8

Thiebaut, F. I., White, S. J., Walsh, A., Klargaard, S. K., Wu, H. C., Rees, G., & Burgess, P. W. (2016). Does Faux Pas Detection in Adult Autism Reflect Differences in Social Cognition or Decision-Making Abilities? *J Autism Dev Disord*, *46*(1), 103–112. https://doi.org/10.1007/s10803-015-2551-1

Timonen-Soivio, L., Vanhala, R., Malm, H., Hinkka-Yli-Salomaki, S., Gissler, M., Brown, A., & Sourander, A. (2016). Brief Report: Syndromes in Autistic Children in a Finnish Birth Cohort. *J Autism Dev Disord*, *46*(8), 2780–2784. https://doi.org/10.1007/s10803-016-2789-2

Tirado, M. J., & Saldana, D. (2016). Readers with Autism Can Produce Inferences, but they Cannot Answer Inferential Questions. *J Autism Dev Disord*, *46*(3), 1025–1037. https://doi.org/10.1007/s10803-015-2648-6

Trevisan, D. A., Bowering, M., & Birmingham, E. (2016). Alexithymia, but not autism spectrum disorder, may be related to the production of emotional facial expressions. *Mol Autism*, *7*, 46. https://doi.org/10.1186/s13229-016-0108-6

Troyb, E., Knoch, K., Herlihy, L., Stevens, M. C., Chen, C. M., Barton, M., … Fein, D. (2016). Restricted and Repetitive Behaviors as Predictors of Outcome in Autism Spectrum Disorders. *J Autism Dev Disord*, *46*(4), 1282–1296. https://doi.org/10.1007/s10803-015-2668-2

Turcotte, P., Mathew, M., Shea, L. L., Brusilovskiy, E., & Nonnemacher, S. L. (2016). Service Needs Across the Lifespan for Individuals with Autism. *J Autism Dev Disord*, *46*(7), 2480–2489. https://doi.org/10.1007/s10803-016-2787-4

Uljarevic, M., Evans, D. W., Alvares, G. A., & Whitehouse, A. J. (2016). Short report: relationship between restricted and repetitive behaviours in children with autism spectrum disorder and their parents. *Mol Autism*, *7*, 29. https://doi.org/10.1186/s13229-016-0091-y

Uljarevic, M., Lane, A., Kelly, A., & Leekam, S. (2016). Sensory subtypes and anxiety in older children and adolescents with autism spectrum disorder. *Autism Res*, *9*(10), 1073–1078. https://doi.org/10.1002/aur.1602

Ure, A. M., Treyvaud, K., Thompson, D. K., Pascoe, L., Roberts, G., Lee, K. J., … Anderson, P. J. (2016). Neonatal brain abnormalities associated with autism spectrum disorder in children born very preterm. *Autism Res*, *9*(5), 543–552. https://doi.org/10.1002/aur.1558

Uzefovsky, F., Allison, C., Smith, P., & Baron-Cohen, S. (2016). Brief Report: The Go/No-Go Task Online: Inhibitory Control Deficits in Autism in a Large Sample. *J Autism Dev Disord*, *46*(8), 2774–2779. https://doi.org/10.1007/s10803-016-2788-3

van Boxtel, J. J., Dapretto, M., & Lu, H. (2016). Intact recognition, but attenuated adaptation, for biological motion in youth with autism spectrum disorder. *Autism Res*, *9*(10), 1103–1113. https://doi.org/10.1002/aur.1595

Van der Hallen, R., Evers, K., Boets, B., Steyaert, J., Noens, I., & Wagemans, J. (2016). Visual Search in ASD: Instructed Versus Spontaneous Local and Global Processing. *J Autism Dev Disord*, *46*(9), 3023–3036. https://doi.org/10.1007/s10803-016-2826-1

Vanmarcke, S., Mullin, C., Van der Hallen, R., Evers, K., Noens, I., Steyaert, J., & Wagemans, J. (2016). In the Eye of the Beholder: Rapid Visual Perception of Real-Life Scenes by Young Adults with and Without ASD. *J Autism Dev Disord*, *46*(8), 2635–2652. https://doi.org/10.1007/s10803-016-2802-9

Vanmarcke, S., Van Der Hallen, R., Evers, K., Noens, I., Steyaert, J., & Wagemans, J. (2016). Ultra-Rapid Categorization of Meaningful Real-Life Scenes in Adults With and Without ASD. *J Autism Dev Disord*, *46*(2), 450–466. https://doi.org/10.1007/s10803-015-2583-6

Virk, J., Liew, Z., Olsen, J., Nohr, E. A., Catov, J. M., & Ritz, B. (2016). Preconceptional and prenatal supplementary folic acid and multivitamin intake and autism spectrum disorders. *Autism*, *20*(6), 710–718. https://doi.org/10.1177/1362361315604076

Vivanti, G., Hocking, D. R., Fanning, P., & Dissanayake, C. (2016). Social affiliation motives modulate spontaneous learning in Williams syndrome but not in autism. *Mol Autism*, *7*(1), 40. https://doi.org/10.1186/s13229-016-0101-0

Vogan, V. M., Morgan, B. R., Leung, R. C., Anagnostou, E., Doyle-Thomas, K., & Taylor, M. J. (2016). Widespread White Matter Differences in Children and Adolescents with Autism

Spectrum Disorder. *J Autism Dev Disord*, *46*(6), 2138–2147. https://doi.org/10.1007/s10803-016-2744-2

Vohra, R., Madhavan, S., & Sambamoorthi, U. (2016). Emergency Department Use Among Adults with Autism Spectrum Disorders (ASD). *J Autism Dev Disord*, *46*(4), 1441–1454. https://doi.org/10.1007/s10803-015-2692-2

Walsh, J. A., Creighton, S. E., & Rutherford, M. D. (2016). Emotion Perception or Social Cognitive Complexity: What Drives Face Processing Deficits in Autism Spectrum Disorder? *J Autism Dev Disord*, *46*(2), 615–623. https://doi.org/10.1007/s10803-015-2606-3

Weiss, J. A., Tint, A., Paquette-Smith, M., & Lunsky, Y. (2016). Perceived self-efficacy in parents of adolescents and adults with autism spectrum disorder. *Autism*, *20*(4), 425–434. https://doi.org/10.1177/1362361315586292

Wenger, T. L., Miller, J. S., DePolo, L. M., de Marchena, A. B., Clements, C. C., Emanuel, B. S., … Schultz, R. T. (2016). 22q11.2 duplication syndrome: elevated rate of autism spectrum disorder and need for medical screening. *Mol Autism*, *7*, 27. https://doi.org/10.1186/s13229-016-0090-z

Whitaker, L., Jones, C. R., Wilkins, A. J., & Roberson, D. (2016). Judging the Intensity of Emotional Expression in Faces: the Effects of Colored Tints on Individuals With Autism Spectrum Disorder. *Autism Res*, *9*(4), 450–459. https://doi.org/10.1002/aur.1506

Wicker, B., Monfardini, E., & Royet, J. P. (2016). Olfactory processing in adults with autism spectrum disorders. *Mol Autism*, *7*, 4. https://doi.org/10.1186/s13229-016-0070-3

Wilson, C. E., Murphy, C. M., McAlonan, G., Robertson, D. M., Spain, D., Hayward, H., … Murphy, D. G. (2016). Does sex influence the diagnostic evaluation of autism spectrum disorder in adults? *Autism*, *20*(7), 808–819. https://doi.org/10.1177/1362361315611381

Wink, L. K., Adams, R., Wang, Z., Klaunig, J. E., Plawecki, M. H., Posey, D. J., … Erickson, C. A. (2016). A randomized placebo-controlled pilot study of N-acetylcysteine in youth with autism spectrum disorder. *Mol Autism*, *7*, 26. https://doi.org/10.1186/s13229-016-0088-6

Woodman, A. C., Smith, L. E., Greenberg, J. S., & Mailick, M. R. (2016). Contextual Factors Predict Patterns of Change in Functioning over 10 Years Among Adolescents and Adults with Autism Spectrum Disorders. *J Autism Dev Disord*, *46*(1), 176–189. https://doi.org/10.1007/s10803-015-2561-z

Yakubova, G., Hughes, E. M., & Shinaberry, M. (2016). Learning with Technology: Video Modeling with Concrete-Representational-Abstract Sequencing for Students with Autism Spectrum Disorder. *J Autism Dev Disord*, *46*(7), 2349–2362. https://doi.org/10.1007/s10803-016-2768-7

Yang, D. Y., Beam, D., Pelphrey, K. A., Abdullahi, S., & Jou, R. J. (2016). Cortical morphological markers in children with autism: a structural magnetic resonance imaging study of thickness, area, volume, and gyrification. *Mol Autism*, *7*, 11. https://doi.org/10.1186/s13229-016-0076-x

Yang, S., Paynter, J. M., & Gilmore, L. (2016). Vineland Adaptive Behavior Scales: II Profile of Young Children with Autism Spectrum Disorder. *J Autism Dev Disord*, *46*(1), 64–73. https://doi.org/10.1007/s10803-015-2543-1

Yin, C. L., Chen, H. I., Li, L. H., Chien, Y. L., Liao, H. M., Chou, M. C., … Gau, S. S. (2016). Genome-wide analysis of copy number variations identifies PARK2 as a candidate gene for autism spectrum disorder. *Mol Autism*, *7*, 23. https://doi.org/10.1186/s13229-016-0087-7

Yoshimura, Y., Kikuchi, M., Hiraishi, H., Hasegawa, C., Takahashi, T., Remijn, G. B., … Kojima, H. (2016). Atypical development of the central auditory system in young children with Autism spectrum disorder. *Autism Res*, *9*(11), 1216–1226. https://doi.org/10.1002/aur.1604

Young, H. E., Falco, R. A., & Hanita, M. (2016). Randomized, Controlled Trial of a Comprehensive Program for Young Students with Autism Spectrum Disorder. *J Autism Dev Disord*, *46*(2), 544–560. https://doi.org/10.1007/s10803-015-2597-0

Zamora, I., Williams, M. E., Higareda, M., Wheeler, B. Y., & Levitt, P. (2016). Brief Report: Recruitment and Retention of Minority Children for Autism Research. *J Autism Dev Disord*, *46*(2), 698–703. https://doi.org/10.1007/s10803-015-2603-6

Zeedyk, S. M., Cohen, S. R., Eisenhower, A., & Blacher, J. (2016). Perceived Social Competence and Loneliness Among Young Children with ASD: Child, Parent and Teacher Reports. *J Autism Dev Disord*, *46*(2), 436–449. https://doi.org/10.1007/s10803-015-2575-6

Zwaigenbaum, L., Bryson, S. E., Brian, J., Smith, I. M., Roberts, W., Szatmari, P., … Vaillancourt, T. (2016). Stability of diagnostic assessment for autism spectrum disorder between 18 and 36 months in a high-risk cohort. *Autism Res*, *9*(7), 790–800. https://doi.org/10.1002/aur.1585
